# Variations in disease burden of laryngeal cancer attributable to alcohol use and smoking in 204 countries or territories, 1990–2019

**DOI:** 10.1186/s12885-021-08814-4

**Published:** 2021-10-07

**Authors:** Qiang-Wei Zhang, Jing-Yuan Wang, Xiao-Feng Qiao, Tong-Li Li, Xin Li

**Affiliations:** 1 Department of Otorhinolaryngology, Shanxi Provincial People’s Hospital Affiliated to Shanxi Medical University, No. 29 of Twin Towers Temple Street, Taiyuan, 030012 Shanxi China; 2 Department of Otolaryngology Head and Neck Surgery, Shanxi Bethuen Hospital, Shanxi Academy of Medical Sciences, Tongji Shanxi Hospital, Third Hospital of Shanxi Medical University, No.99 Longcheng Street, Taiyuan, 030032 Shanxi China

**Keywords:** Laryngeal cancer, Mortality, Disease burden, Alcohol, Tobacco

## Abstract

**Background:**

Alcohol consumption and smoking are the leading risk factors for laryngeal cancer (LC). Understanding the variations in disease burden of LC attributable to alcohol use and smoking is critical for LC prevention.

**Methods:**

Disease burden data of LC were retrieved from the Global Burden of Disease Study 2019. We used estimated average percentage change (EAPC) to measure the temporal trends of the age-standardized mortality rate (ASMR) of LC.

**Results:**

Globally, while the ASMR of LC decreased by 1.49% (95% CI, 1.41–1.57%) per year between 1990 and 2019, the number of deaths from LC has increased 41.0% to 123.4 thousand in 2019. In 2019, 19.4 and 63.5% of total LC-related deaths were attributable to alcohol use and smoking worldwide, respectively. The ASMR of alcohol- and smoking-related LC decreased by 1.78 and 1.93% per year, whereas the corresponding death number has increased 29.2 and 25.1% during this period, respectively. The decreasing trend was more pronounced in developed countries. In some developing countries, such as Guinea and Mongolia, the LC mortality has shown an unfavorable trend.

**Conclusion:**

The ubiquitous decrease in LC mortality was largely attributed to the smoking control and highlighted the importance of smoking control policies. However, the disease burden of LC remained in increase and more effective strategies are needed to combat the global increase of alcohol consumption.

**Supplementary Information:**

The online version contains supplementary material available at 10.1186/s12885-021-08814-4.

## Introduction

Laryngeal cancer (LC) remains one of the most common tumors of the respiratory tract [1] and has a low survival at late stages [2]. In 2018, it is reported that a total of 177 thousand and 95 thousand new cases of laryngeal cancer and its related deaths were occurred worldwide, respectively [3]. The risk factors for LC have been extensively studied. The most significant of these are smoking and alcohol use [4, 5]. Smoking has been shown to associate with the carcinogenesis of LC in a dose-dependent manner, with a risk for smokers that is 10 to 15 times higher than the risk for nonsmokers, and the heaviest smokers have as high as a 30 times greater risk [6]. Likewise, previous studies have shown a linear relationship between alcohol consumption and risk of LC [7]. Although exposure to several other environmental factors, such as asbestos, polycyclic aromatic hydrocarbons, and textile dust [8], is thought to increase the risk of LC, these factors contributed less to the disease burden of LC when compared with smoking and alcohol use. In other word, variations in the prevalence of smoking and alcohol consumption largely influenced the temporal trends of LC morbidity and mortality. Knowing the contributions of smoking and alcohol use to the LC disease burden during the last decades is therefore critical for LC prevention.

In this regard, the Global Burden of Disease (GBD) study, which integrated sparse data in real world and then processed them using advanced modeling strategies, provided us an opportunity to learn the LC disease burden from multiple facets [9]. In the current study, using the data of GBD study 2019, we described the variations in disease burden of LC attributable to smoking and alcohol use at the global, regional, and national levels. Our findings are important to learn the current disease burden of LC and to update the prevention strategies.

## Materials and methods

### Study data

#### Data of LC disease burden

GBD 2019 provides a rules-based synthesis of the available evidence on levels and trends in health outcomes, a diverse set of risk factors, and health system responses [9]. GBD 2019 incorporates data from 281,586 sources and provides more than 3.5 billion estimates of health outcome and health system measures of interest for global, national, and subnational policy dialogue. Herein, we retrieved the data of LC disease burden from the GBD study online database [10]. Specifically, we collected the data of death number, age-standardized mortality rate (ASMR), and disability-adjusted life years (DALYs) of LC. One DALY can be thought of as one lost year of “healthy” life. The sum of these DALYs across the population, or the burden of disease, can be thought of as a measurement of the gap between current health status and an ideal health situation where the entire population lives to an advanced age, free of disease and disability. These data were archived by sex, age, calendar year, and region. Herein, we retrieved the data at global, regional, and national levels. Data from 5 regions according to socio-demographical indexes (SDI) and from 21 GBD regions according to geography were available. SDI is a composite indicator of development status strongly correlated with health outcomes. It is the geometric mean of 0 to 1 indices of total fertility rate under the age of 25, mean education for those ages 15 and older, and lag distributed income per capita. As a composite, a location with an SDI of 0 would have a theoretical minimum level of development relevant to health, while a location with an SDI of 1 would have a theoretical maximum level [11]. Additionally, data from a total of 204 countries or territories were available.

The procedure of cancer data processing in GBD study has been detailed previously [12]. For LC, briefly, the mortality data were collected from vital registries and cancer registries identifying by the ICD-10 codes of C32-C32.9 and ICD-9 codes of 161–161.9. In the GBD study, a total of 5236 death sources were available for LC. These data were then modeled by Cause of Death Ensemble modelling (CODEm), which is the framework used to model most cause-specific death rates in the GBD study. For locations that lack of cancer data, the estimates rely either on predictive covariates or trends from neighboring locations [13]. GBD study also provides 84 risk-outcome pairs to estimate disease burden caused by behavioral, environmental and occupational, and metabolic risks or clusters of risks [14]. In this study, we retrieved the mortality data of LC attributable to smoking and alcohol use.

#### Data of smoking and alcohol use

We also retrieved the prevalence data in smoking and alcohol consumption volume (per capita in liters of pure alcohol in people aged ≥15 years) at national level from the World Health Organization (www.who.int/gho/en). The smoking prevalence data were available at 189 countries or territories and covered from year of 1980 to 2012. The alcohol use data were available at 193 countries or territories and covered from year of 1980 to 2015.

### Statistical analysis

As previously reported, we used estimated average percentage change (EAPC) to quantify the LC ASMR between 1990 and 2019 [15, 16]. The EAPC was also applied to quantify the temporal trends of smoking prevalence and alcohol consumption. The EAPC can be calculated from the linear regression model, that is y = α + βx + ɛ, where y = ln (ASMR), x = calendar year, and ɛ denotes the random deviation. Pearson correlation test was used to assess the correlation between temporal trends of LC ASMR and that of smoking prevalence and alcohol use. All statistical tests were analyzed using the R program (R core team, version 3.6.3, Vienna, Austria). A *P* value < 0.05 was considered statistically significant.

### Sensitivity analysis

To ensure the robustness of our results, we conducted a sensitivity analysis. We further incorporated an uncertainty term into the linear regression model as the weight, that is y = α + βx + ɛ + w. The uncertainty term “w” was calculated by 1/((U-L)/L), where U and L represent the upper and lower limits of the estimates of ASMR, respectively. The wider the uncertainty interval, the lower the weight. The coefficient “β”, intercept term “α” and random deviation ɛ can be calculated by the following formulas:

$$ \upbeta =\frac{\sum_{i=1}^n\left({x}_i-\overline{x}\right)\left({y}_i-\overline{y}\right)}{\sum_{i=1}^n{\left({x}_i-\overline{x}\right)}^2},\kern0.36em \upalpha =\overline{y}-\beta \overline{x},\mathrm{and}\kern0.24em \varepsilon ={y}_i-{\hat{y}}_l. $$

## Results

### Disease burden of laryngeal cancer attributable to all causes

**Fig. 1:**
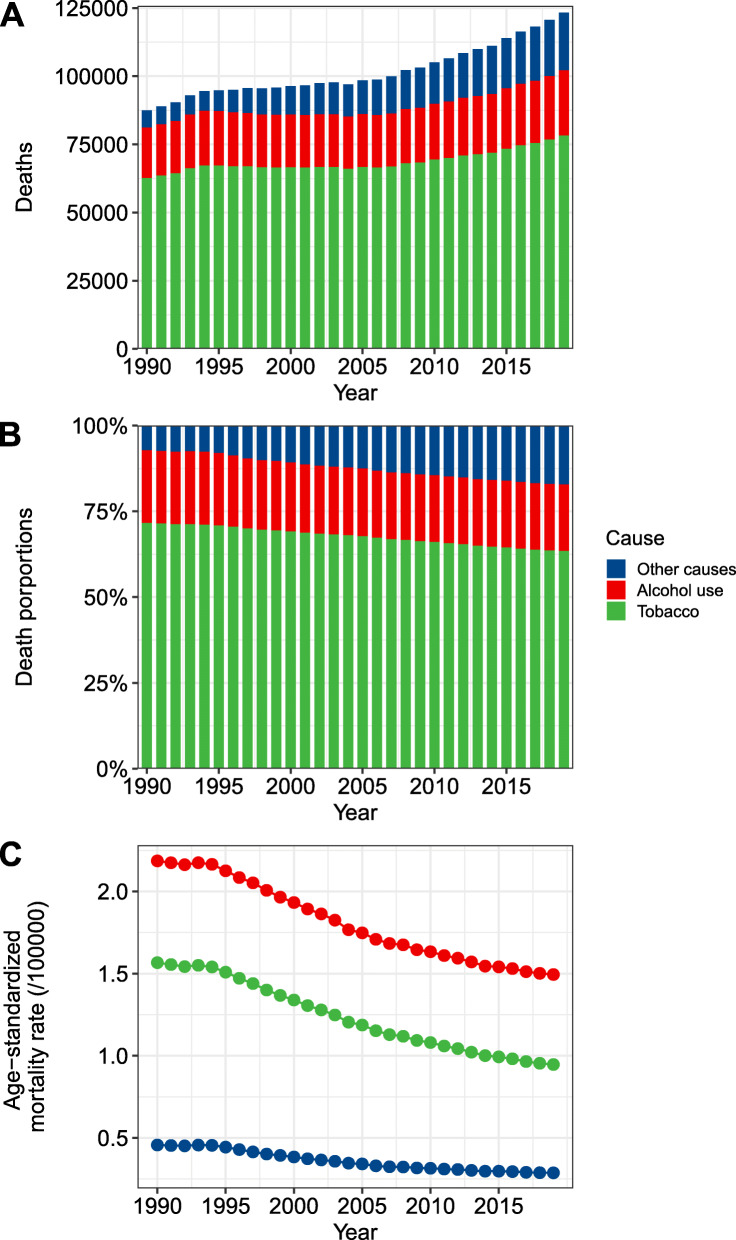
The changing trend of disease burden of laryngeal cancer (LC) at the global level between 1990 and 2019 (**A**. age-standardized mortality rate of LC attributable to alcohol use, smoking and other causes; **B**. absolute number of LC-related deaths attributable to alcohol use, smoking and other causes; **C**. proportion of death number from LC attributable to alcohol use, smoking and other causes)

Globally, while the ASMR of LC decreased by 1.49% (95% CI, 1.41–1.57%) per year between 1990 and 2019 (Fig. 1A), the number of deaths from LC has increased 41.0% to 123.4 thousand in 2019 (Fig. 1B; Table 1). Correspondingly, the DALYs of LC increased from 2.5 million person-years (pys) to 3.3 million pys in the same period (Supplementary Fig. S1). More than 85% of total LC deaths were occurred in males. Males experienced a more remarkable decrease in LC ASMR than females (Table 1).

**Table 1:** The mortality of larynx cancer in 1990 and 2019 and the temporal trends between 1990 and 2019, by sex, risk factor, SDI region, and GBD region

	All causes					Alcohol use					Tobacco use				
	1990		2019		1990–2019	1990		2019		1990–2019	1990		2019		1990–2019
	Death number (×1000)	ASMR (/10^5^)	Death number (×1000)	ASMR (/10^5^)	EAPC (95% CI)	Death number (×1000)	ASMR (/10^5^)	Death number (×1000)	ASMR (/10^5^)	EAPC (95% CI)	Death number (×1000)	ASMR (/10^5^)	Death number (×1000)	ASMR (/10^5^)	EAPC (95% CI)
Global	87.5	2.19	123.4	1.49	−1.49 (−1.57, − 1.41)	18.5	0.46	23.9	0.29	−1.78 (− 1.90, − 1.66)	62.6	1.57	78.3	0.95	−1.93 (−2.00, − 1.85)
Sex															
Male	75.9	4.15	105.6	2.74	−2.19 (− 2.34, −1.98)	17.7	0.95	22.3	0.59	−2.32 (− 2.56, − 2.01)	58.6	3.21	73.4	1.91	−2.43 (− 2.54, − 2.31)
Female	11.6	0.54	17.8	0.41	−1.22 (− 1.54, − 0.89)	0.8	0.04	1.6	0.02	−1.42 (− 1.76, − 1.09)	4.0	0.19	4.9	0.11	− 1.38 (− 1.53, − 1.23)
SDI regions															
High	15.7	1.53	14.6	0.79	−2.56 (− 2.71, − 2.41)	5.1	0.50	4.2	0.23	−2.78 (− 2.94, − 2.62)	12.5	1.21	10.4	0.56	− 2.92 (− 3.07, − 2.78)
High-middle	29.6	2.72	30.4	1.48	−2.47 (− 2.60, − 2.34)	8.8	0.80	8.2	0.40	−2.79 (− 2.95, − 2.63)	23.1	2.11	21.9	1.06	− 2.76 (− 2.90, − 2.62)
Middle	18.3	1.81	34.9	1.42	−0.86 (− 0.91, − 0.81)	2.6	0.25	6.3	0.25	0.01 (− 0.13, 0.15)	12.2	1.21	21.9	0.89	−1.10 (− 1.15, − 1.05)
Low-middle	17.5	2.91	32.1	2.34	−0.80 (− 0.88, − 0.72)	1.5	0.23	3.9	0.28	0.75 (0.55, 0.96)	11.6	1.95	18.8	1.38	−1.22 (− 1.29, − 1.14)
Low	6.2	2.59	11.2	2.16	−0.70 (− 0.77, − 0.64)	0.5	0.21	1.2	0.22	0.14 (0.01, 0.29)	3.2	1.36	5.2	1.02	−1.07 (− 1.16, − 0.98)
GBD region															
Andean Latin America	0.2	1.26	0.4	0.73	−1.92 (−2.17, − 1.68)	< 100	0.17	0.1	0.10	−1.53 (− 1.90, − 1.16)	0.1	0.46	0.1	0.24	−2.23 (− 2.45, − 2.02)
Australasia	0.3	1.24	0.3	0.59	−2.79 (− 2.92, − 2.67)	0.1	0.46	0.1	0.20	−2.99 (−3.13, − 2.86)	0.2	0.87	0.1	0.29	−3.97 (−4.11, − 3.84)
Caribbean	0.8	2.99	1.5	2.97	0.05 (−0.04, 0.14)	0.1	0.52	0.3	0.58	−2.02 (− 2.24, −1.80)	0.5	2.03	1.0	1.93	−1.71 (− 1.81, − 1.62)
Central Asia	1.5	3.05	1.3	1.77	−2.15 (− 2.33, − 1.97)	0.3	0.59	0.3	0.32	− 2.40 (− 2.54, − 2.27)	1.1	2.13	0.9	1.14	− 2.37 (− 2.50, − 2.23)
Central Europe	5.5	3.68	5.3	2.58	− 1.39 (− 1.49, − 1.29)	2.0	1.31	1.8	0.92	−1.35 (− 1.45, − 1.26)	4.4	2.95	4.0	1.96	− 1.56 (− 1.66, − 1.45)
Central Latin America	1.6	2.00	2.7	1.15	− 2.35 (− 2.48, − 2.22)	0.3	0.35	0.5	0.20	− 2.36 (− 2.50, − 2.21)	0.9	1.16	1.2	0.52	−3.23 (− 3.38, − 3.08)
Central Sub-Saharan Africa	0.4	1.72	0.7	1.33	−0.96 (− 1.05, − 0.87)	0.1	0.22	0.1	0.20	0.02 (−0.49, 0.52)	0.2	0.66	0.3	0.50	−0.95 (−1.16, − 0.75)
East Asia	11.9	1.39	20.9	1.01	−0.97 (−1.07, − 0.87)	2.3	0.26	4.8	0.23	−0.40 (− 0.61, − 0.19)	8.5	1.00	15.8	0.75	− 0.86 (− 0.96, − 0.75)
Eastern Europe	9.5	3.32	6.6	1.93	−2.77 (−3.12, − 2.42)	3.0	1.06	2.2	0.65	−2.56 (− 2.96, − 2.15)	7.6	2.64	5.2	1.51	−2.84 (−3.20, − 2.47)
Eastern Sub-Saharan Africa	1.3	1.64	2.0	1.22	−1.13 (− 1.18, − 1.08)	0.2	0.26	0.4	0.21	−0.78 (− 0.87, − 0.69)	0.5	0.64	0.7	0.45	−1.28 (− 1.32, − 1.24)
High-income Asia Pacific	1.8	0.89	1.8	0.37	−3.67 (− 3.94, − 3.41)	0.5	0.24	0.4	0.09	−3.86 (−4.17, − 3.55)	1.4	0.71	1.3	0.27	−4.03 (− 4.30, − 3.76)
High-income North America	4.7	1.36	5.4	0.87	−1.89 (−2.01, − 1.76)	1.1	0.32	1.3	0.22	−1.51 (− 1.63, − 1.38)	3.7	1.09	3.9	0.62	−2.26 (− 2.39, − 2.14)
North Africa and Middle East	4.1	2.42	7.6	1.82	−0.97 (−1.08, − 0.87)	0.2	0.08	0.3	0.06	−1.24 (− 1.34, − 1.14)	2.9	1.69	5.1	1.22	−1.10 (− 1.20, − 1.01)
Oceania	0.0	0.97	0.1	0.85	−0.44 (− 0.46, − 0.42)	< 100	0.06	< 100	0.05	−0.59 (− 0.90, − 0.29)	0.0	0.54	0.0	0.42	− 0.93 (− 0.97, − 0.89)
South Asia	21.5	3.76	39.9	2.80	−1.15 (− 1.24, − 1.06)	1.6	0.26	4.5	0.31	0.64 (0.37, 0.90)	13.7	2.46	21.7	1.55	−1.70 (− 1.78, − 1.62)
Southeast Asia	3.6	1.42	7.1	1.20	−0.64 (− 0.72, − 0.56)	0.3	0.13	1.3	0.21	1.86 (1.68, 2.04)	2.4	0.99	4.8	0.81	−0.78 (− 0.87, − 0.70)
Southern Latin America	1.4	2.95	1.4	1.64	−2.36 (−2.53, −2.19)	0.5	1.07	0.4	0.52	−2.88 (−3.04, − 2.72)	1.0	2.08	0.9	1.03	−2.78 (− 2.97, − 2.58)
Southern Sub-Saharan Africa	0.6	1.96	0.9	1.57	−1.05 (−1.52, −0.59)	0.1	0.49	0.2	0.36	−1.50 (−2.02, −0.99)	0.4	1.36	0.5	0.89	−1.83 (−2.29, − 1.37)
Tropical Latin America	2.8	3.00	5.5	2.26	−0.94 (−1.01, − 0.86)	0.5	0.54	1.2	0.47	−0.52 (− 0.77, − 0.26)	2.2	2.36	3.2	1.30	−2.07 (− 2.22, − 1.92)
Western Europe	13.2	2.38	9.7	1.11	−2.79 (− 2.92, − 2.67)	5.1	0.92	3.3	0.38	−3.16 (−3.29, − 3.04)	10.6	1.91	6.9	0.80	−3.16 (− 3.28, − 3.05)
Western Sub-Saharan Africa	1.1	1.28	2.3	1.28	0.23 (0.09, 0.37)	0.2	0.21	0.4	0.23	0.60 (0.41, 0.79)	0.4	0.42	0.7	0.39	−0.03 (−0.16, 0.11)

*SDI* socio-demographical index; *GBD* global burden of disease; *ASMR* age-standardized mortality rate; *EAPC* estimated average percentage change

**Fig. 2:**
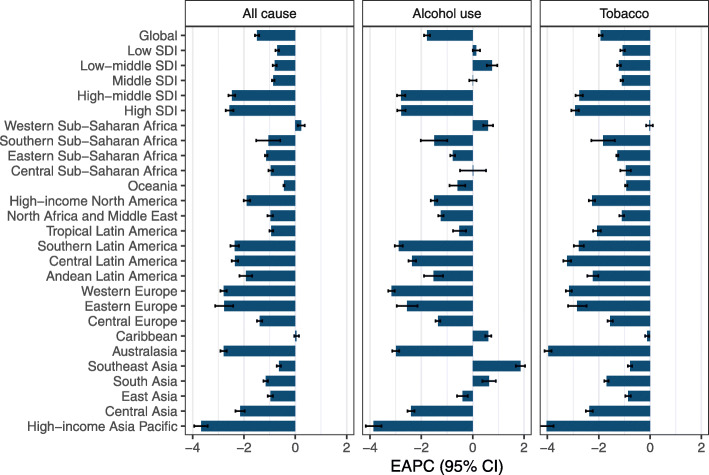
The changing trends in age-standardized mortality rate (ASMR) of laryngeal cancer attributable to all cause, alcohol use, and smoking in 1990–2019 at the global and regional levels. The changing trends were quantified by estimated average percentage change (EAPC). The ASMR was deemed to be increased if the EAPC estimate and the lower boundary of its 95% CI were both > 0. In contrast, the ASMR was decreased if the EAPC estimate and the upper boundary of its 95% CI were both < 0. Otherwise, the ASMR was deemed to be stable over time

The ASMR decreased in all SDI-regions (Table 1). In contrast, the death number and DALYs were increased in all regions, with an exception was found in high-SDI region. Low-SDI region experienced the most pronounced increase in the death number and DALYs of LC, followed by low-middle SDI region (Table 1). We found that only one GBD region, Western Sub-Saharan Africa, showed a significant increase in LC ASMR (EAPC = 0.23, 95% CI, 0.09, 0.37) (Fig. 2; Table 1). The most significant decrease was observed in High−income Asia Pacific, followed by Australasia and Western Europe. The death number and DALYs of LC were decreased in six GBD regions (Table 1; Supplementary Fig. S2).

**Fig. 3:**
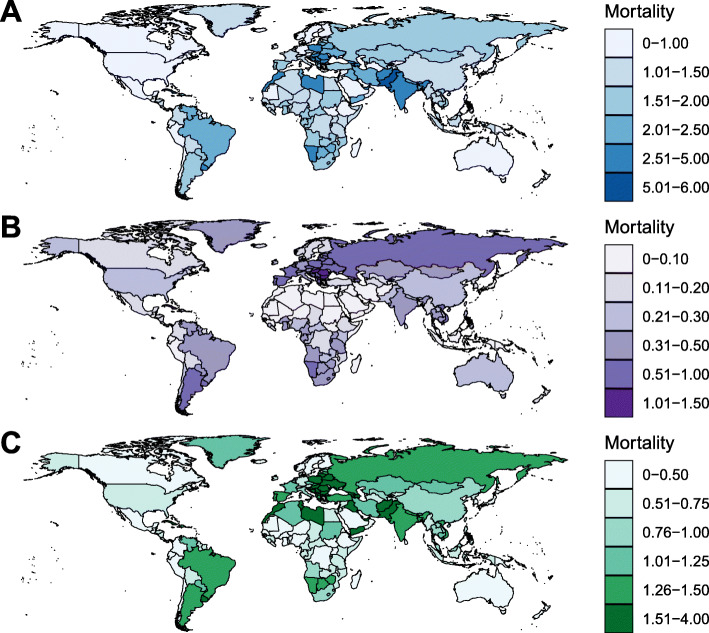
The age-standardized mortality rate (per 100,000) of laryngeal cancer attributable to (**A**) all-cause, (**B**) alcohol use, and (**C**) smoking in 2019. The deeper the color, the higher the mortality rate

**Fig. 4:**
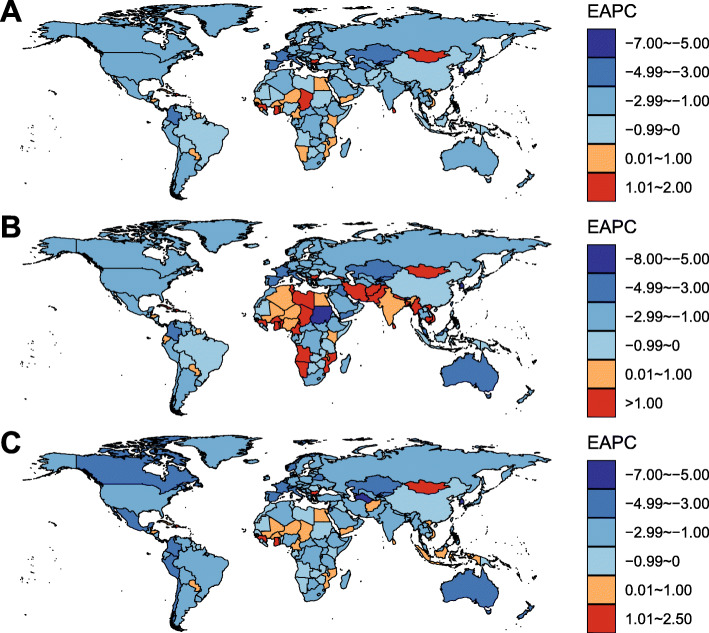
The changing trends in age-standardized mortality rate (ASMR) of laryngeal cancer attributable to (**A**) all-cause, (**B**) alcohol use, and (**C**) smoking at the national level between 1990 and 2019

At the national level, in 2019, the ASMR of LC varied nearly 20 times from country to country. The highest ASMR of LC was found in Pakistan (ASMR = 5.75/100,000) (Fig. 3A). Between 1990 and 2019, there were 29 countries or territories, which were not advanced in economy, experienced a significant increase in LC ASMR. A total of 162 countries or territories experienced a significant decrease in LC ASMR (Fig. 4A; Supplementary Table S1). The greatest decrease was observed in most developed countries. The highest number of deaths from LC was found in India, followed by China and Pakistan.

### Disease burden of laryngeal cancer attributable to alcohol use

In 2019, 19.4% of total LC-related deaths were attributable to alcohol use worldwide (Fig. 1C). The ASMR of alcohol-related LC decreased by 1.78% per year (Fig. 1A), whereas the death number has increased 29.2% during the study period (Fig. 1B; Table 1). The corresponding DALYs increased from 0.54 million pys in 1990 to 0.66 million pys in 2019. At the GBD regional level, 4 and 16 regions have shown a significantly increasing and decreasing trend in alcohol-related LC ASMR, respectively (Table 1; Fig. 2). The greatest increase was observed in Southeast Asia (EAPC = 1.86, 95% CI 1.68–2.04). The most pronounced decrease was found in High-income Asia Pacific (EAPC = − 3.86, 95% CI, − 4.17, − 3.55). At the national level, the highest ASMR of alcohol-related LC was found in Montenegro (1.45/100,000) in 2019 (Fig. 3B). Between 1990 and 2019, a total of 60 and 133 countries or territories experienced a significant increase and decrease in the ASMR of alcohol-related LC, respectively (Fig. 4B; Supplementary Table S1). The greatest increase was detected in Vietnam (EAPC = 9.64, 95% CI, 8.80–10.69). The most remarkable decrease was found in Bahrain (EAPC = − 7.05, 95% CI, − 7.63, − 6.47).

### Disease burden of laryngeal cancer attributable to smoking

In 2019, 63.5% of total LC-related deaths could be attributed to smoking (Fig. 1C). Although the ASMR of smoking-related LC decreased by 1.93% per year between 1990 and 2019 (Fig. 1A), the absolute death number has increased 25.1% during this period (Fig. 1B; Table 1). The decreasing trend in the ASMR was consistent in both sexes and in all GBD regions. An exception was found in Western Sub-Saharan Africa (Fig. 2). At the national level, the highest ASMR of smoking-related LC was found in Seychelles (3.76/100,000) in 2019 (Fig. 3C). From 1990 to 2019, there were only 27 countries or territories have shown a significant increasing trend in the ASMR of smoking-related LC (Fig. 4C; Supplementary Table S1). The greatest increase was detected in Mongolia (EAPC = 2.21, 95% CI, 1.82–2.59). In contrast, there were 168 countries or territories experienced a significant decrease in the ASMR of smoking-related LC, with the most pronounced decrease was found in South Korea (EAPC = − 6.51, 95% CI, − 7.12, − 5.89). The absolute death number and the corresponding DALYs of smoking-related LC were highest in India, followed by China and Pakistan. No contradictory result was found in the sensitivity analysis (Supplementary Table S2). The proportion of LC-related deaths that were associated with risk factors other than smoking and alcohol use increased from 7.3% in 1990 to 17.1% in 2019, connoting approximately 21.1 thousand deaths worldwide. The ASMR of LC attributable to other causes experienced a significantly decrease in 1990–2019 (EAPC = − 1.13, 95% CI, − 0.94, − 1.34).

### Associations of temporal trends in LC ASMR with smoking prevalence, alcohol use, and SDI value

**Fig. 5:**
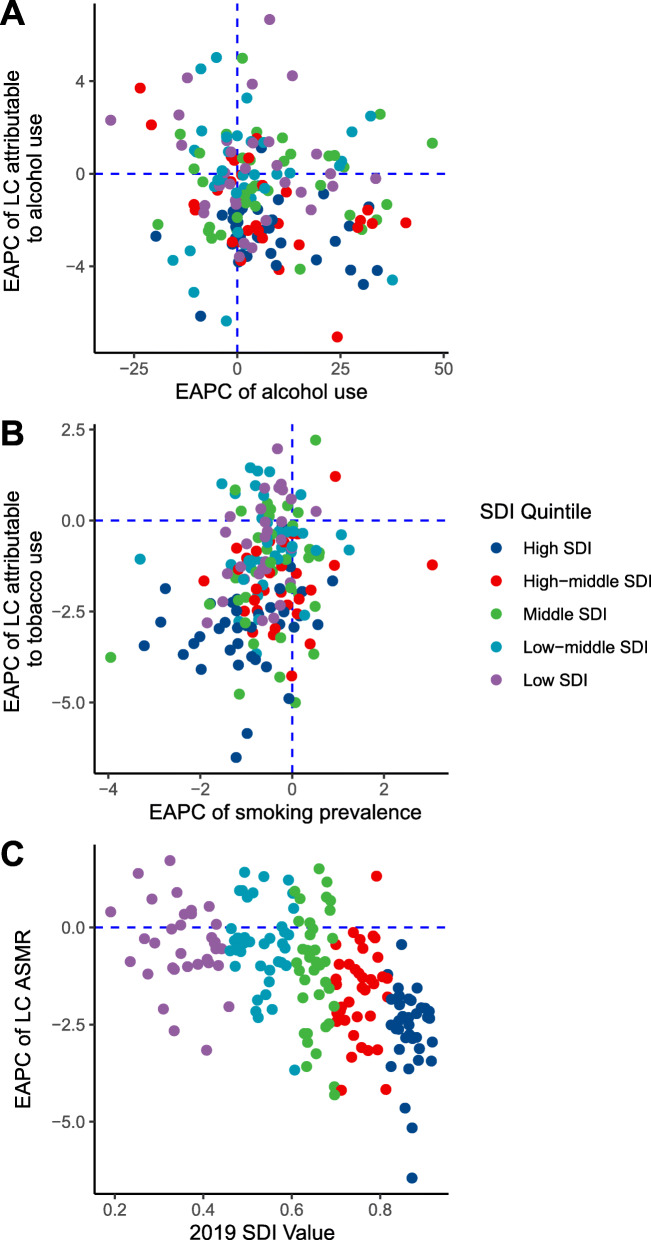
The influential factors for estimated average percentage change (EAPC) of age-standardized mortality rate (ASMR) of laryngeal cancer (LC). (**A**. association between EAPC of alcohol use-related LC mortality rate and EAPC of alcohol use; **B**. association between EAPC of smoking-related LC mortality rate and EAPC of smoking prevalence; **C**. association between EAPC of LC mortality rate and national socio-demographical index values in 2019

Between 1980 and 2015, we found that 138 and 56 countries or territories experienced a significant increase and decrease in the volume of alcohol consumption, respectively (Supplementary Table S3). Between 1980 and 2012, there were a total of 29 countries, such as Afghanistan and Saudi Arabia, experienced a significant increase in smoking prevalence (Supplementary Table S4). In contrast, more than 70% of total countries experienced a significant decrease in smoking prevalence. No significant association was detected between the temporal trends of alcohol use and that of the alcohol-related LC mortality rate (*ρ* = − 0.14, *P* = 0.06) (Fig. 5A). A significantly positive correlation was found between the temporal trends of smoking prevalence and that of the smoking-related LC mortality (*ρ* = 0.26, *P* < 0.001) (Fig. 5B). Additionally, we found a significantly negative correlation between the national SDI values and the temporal trends of LC ASMR (*ρ* = − 0.54, *P* < 0.001). The higher the SDI value, the larger the decreasing magnitude of LC ASMR (Fig. 5C).

## Discussion

In this large-scale population-based study, we reported that LC mortality rate has decreased at the global level and in most countries over the past three decades, although the disease burden of LC remained increasing. Countries with high socio-demographical level had a larger magnitude in the decrease of LC mortality than those with low socio-demographical level. The decreasing trend was mainly driven by the decrease in the LC attributable to smoking. In some developing regions, we also observed an unfavorable trend of LC mortality. The ubiquitous increase of alcohol consumption might result in a reverse of LC mortality in the future, if no intervention was introduced. Our results were in line with that of a previous study, in which the authors analyzed the variations in LC disease burden between 1990 and 2017 [17]. However, different to the previous study that provided a comprehensive description of LC disease burden, we provided more focuses on the most common risk factors of LC, that are alcohol use and smoking. Our findings have implications for the update of prevention strategies of LC in future.

Alcohol drinking and smoking, especially the latter, are the most dominant risk factors for LC [18, 19]. In this study, we found that alcohol use and smoking have contributed to more than 80% of the total LC deaths worldwide. The risk factor-specific proportion of LC-related deaths was highly heterogeneous across the world. For example, in Europe, more than 30% of total LC-related deaths were attributed to alcohol use. This proportion was significantly higher than the world average and was consistent with the high level of alcohol consumption in this region [20, 21]. The heterogeneity not only reveals the difference in risk factor distribution, but also highlights the priority of LC prevention strategies in different countries.

In our study, we found that the absolute number and DALYs of LC were increased at the global level and in most countries. The increase of LC disease burden might be largely ascribed to the population expansion and increasingly ageing population [22]. Fortunately, we observed a ubiquitous decrease in the ASMR of LC worldwide, which was mainly driven by the decrease of smoking-related LC. Over the last decades, enormous efforts have been made to combat smoking [23, 24]. For example, the WHO Framework Convention on Tobacco Control is one of the biggest public health campaigns to protect present and future generations from the devastating health, economic, social and environmental impact of tobacco [25]. Owing to these endeavors, we have seen a significant decrease in smoking prevalence in many countries [26, 27], as shown in our study. Of note is that the persistent increase in the number of smokers worldwide, more endeavors are therefore needed to help smokers to quit and more importantly to protect youth from tobacco use [28]. According to a recent study, the global prevalence of tobacco use among adolescents aged 13–15 years was substantial [29]. This result suggests that we also have a long way to go in the combat with tobacco, which is critical for the prevention of LC.

Alcohol use is another well-determined risk factor of LC, although its contribution to LC is much lower than that of the tobacco use. In our study, while we observed a significant decrease in the alcohol-related LC mortality rate, we also detected a remarkable increase in the alcohol consumption volume in most countries during the last four decades. A recent modeling study reported that the global adult per-capita alcohol consumption increased from 5.9 L to 6.5 L between 1990 and 2017, and is forecasted to reach 7.6 L by 2030 [21]. This unexpected increase might indicate that alcohol will become the leading risk factor for LC in the next decades. In this regard, more effective policies, such as increasing the alcohol tax and strictly forbidding alcohol sale to adolescents, are urgently needed.

The decrease of LC mortality rate was also partly attributed to the development of clinical treatments [30]. In this study, we found that the developed countries with advanced medical levels and more complete medical facilities experienced a more significant decrease in LC mortality rate than the developing countries. However, we have to bear in mind that LC is currently uncurable but preventable. Developing advanced clinical strategies to prolong the survival time of LC patients is important to reduce the LC disease burden. More importantly, preventing the LC onset through alcohol and tobacco control is indeed the fundamental strategy to reduce the LC disease burden. The changing trend of LC disease burden was consistent with that of all cancers worldwide [13]: absolute number increased whereas age-standardized rate decreased. However, between 1990 and 2019, both the mortality rate and DALY of all-cause related deaths were shown a decreasing trend, regardless of the population ageing and growth [12]. These trends suggested that cancers including LC deserve more primacy in future scheme of disease prevention.

The major limitation of our study is that the data of GBD study are results of mathematically models rather than the surveillance data itself. This limitation was largely due to the lack of cancer data in many locations. However, we have to acknowledge that the modeling estimate is a feasible approach to assess the disease burden in regions lacking high-quality registry data and the key principle of GBD study is to take advantage of all relevant data sources. The models used in GBD study, namely the CODEm and DisMod-MR, were deemed to be modelling tools faired best as they fulfilled most of model quality criteria and were specially designed to deal with the diversity of data [31]. Moreover, most GLOBOCAN estimates fell within the 95% uncertainty intervals of the GBD estimates [32], suggesting the robustness of the GBD modeling estimates. In our sensitivity analysis incorporating the uncertainty of GBD estimates, we did not find any contradictory results when compared to the main results. Of note is that the uncertainty of GBD estimates were also obtained from the GBD models. Despite the broad inclusion of different types of data, for certain locations that have neither of these data sources available, the estimates rely either on predictive covariates or trends from neighboring locations. Also, the definition and ascertainment for cause of deaths remains a limitation, which requires further validation. Additionally, the modeling strategies in GBD study lack usability for the general user because of unavailability of sufficient technical detail and customized packages in standard statistical software such as R, SAS, and STATA.

In conclusion, the global LC mortality rate was decreased in the past three decades. This decrease was largely attributed to the smoking control and highlighted the importance of smoking control policies. Of note is the global increase of alcohol consumption, which might result in an unfavorable trend in the LC mortality rate. More effective strategies are therefore warranted to forbid alcohol use.

## Supplementary Information

**Additional file 1.**

## Data Availability

The datasets generated and/or analyzed during the current study are available in the GBD study 2019 online repository, [http://ghdx.healthdata.org/gbd-2017].
