# Supplementary material for: Variations in disease burden of laryngeal cancer attributable to alcohol use and smoking in 204 countries or territories, 1990–2019
Source: BMC Cancer. 2021 Oct 7;21:1082. doi: 10.1186/s12885-021-08814-4 (PMC8496083; doi:10.1186/s12885-021-08814-4)
Supplement: Supplementary file 1 — Additional file 1. [file 12885_2021_8814_MOESM1_ESM.docx]

**Supplementary materials**

**Variations in disease burden of laryngeal cancer attributable to alcohol use and smoking in 204 countries or territories, 1990-2019**

Table Content

[Figure S1. The changing trend of global DALYs of laryngeal cancer attributable to different risk factors, 1990-2019. 2](#_Toc81298931)

[Figure S2. The changing trend of regional DALYs of laryngeal cancer between 1990 and 2019. 3](#_Toc81298932)

[Table S1. The estimate average percentage changes (EAPCs) in age-standardized mortality rate of laryngeal cancer, 1990-2019, by location and risk factor. 4](#_Toc81298933)

[Table S2. The estimate average percentage changes (EAPCs) in age-standardized mortality rate of laryngeal cancer, 1990-2019, by location and risk factor in the sensitivity analysis. 13](#_Toc81298934)

[Table S3. The temporal trends of alcohol consumption at the national level. 24](#_Toc81298935)

[Table S4. The temporal trends of smoking prevalence at the national level. 27](#_Toc81298936)

Figure S1. The changing trend of global DALYs of laryngeal cancer attributable to different risk factors, 1990-2019.


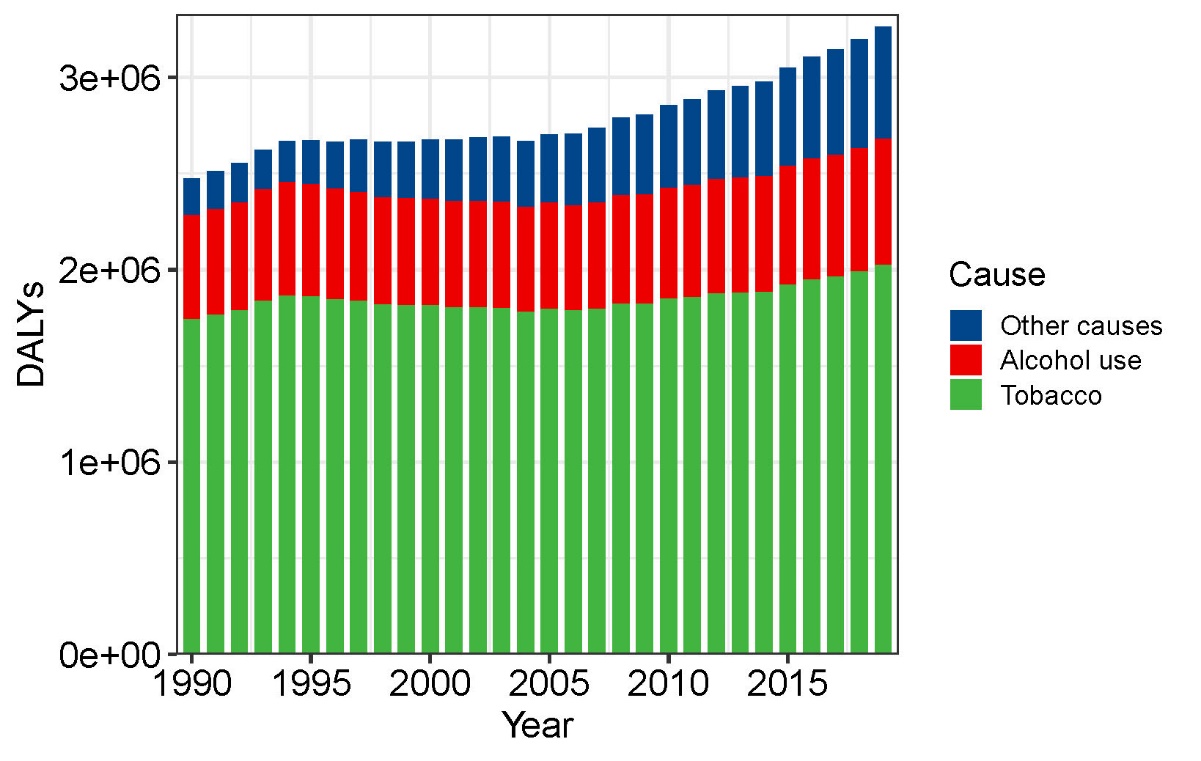


Figure S2. The changing trend of regional DALYs of laryngeal cancer between 1990 and 2019.

(A, all-cause related laryngeal cancer; B, alcohol-related laryngeal cancer; C, tobacco-related laryngeal cancer)


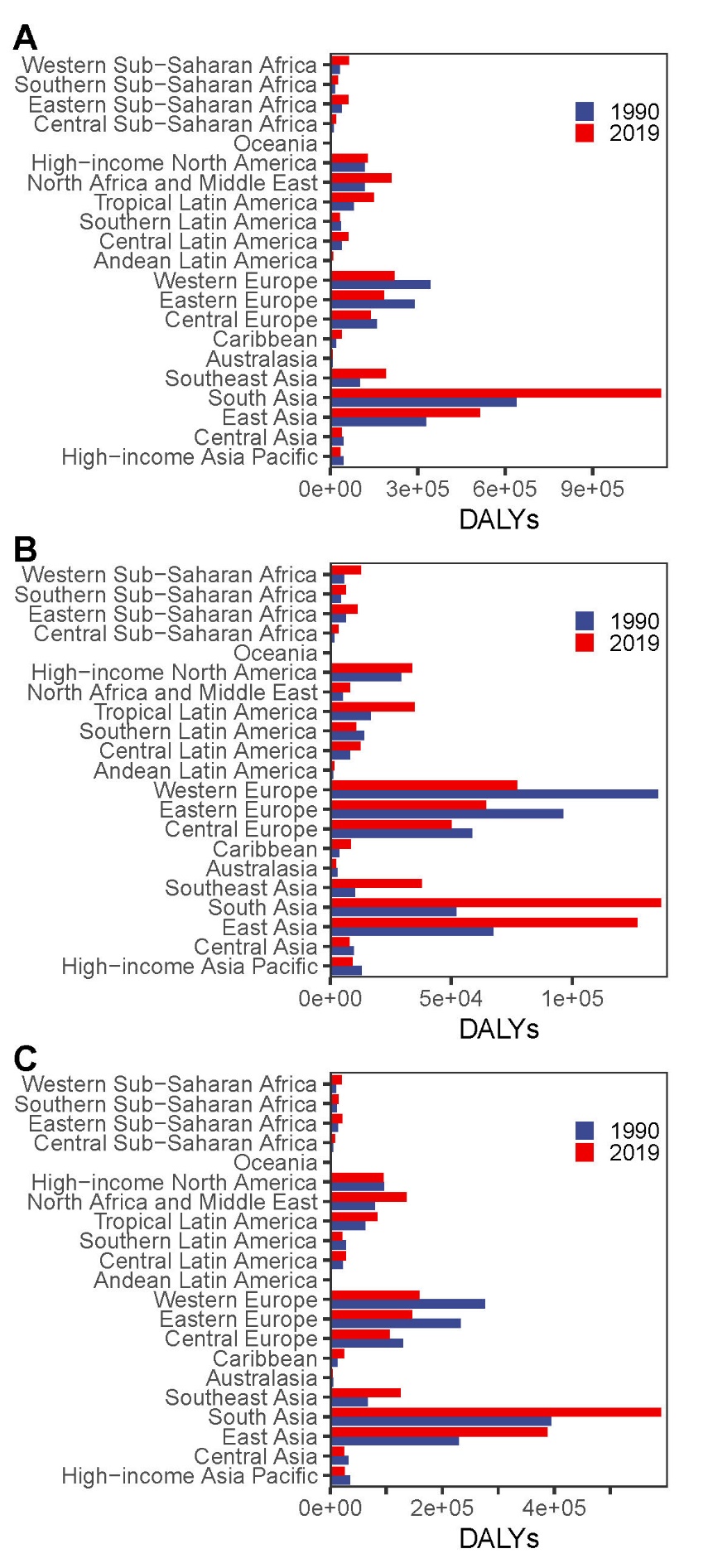


Table S1. The estimate average percentage changes (EAPCs) in age-standardized mortality rate of laryngeal cancer, 1990-2019, by location and risk factor.

| Location name | Risk factors | EAPCs | 95% CI | | P value |
| --- | --- | --- | --- | --- | --- |
| Afghanistan | Alcohol use | 3.87 | 3.3 | 4.45 | <0.001 |
| Afghanistan | All cause | -0.4 | -0.55 | -0.25 | <0.001 |
| Afghanistan | Smoking | 0.25 | 0 | 0.49 | 0.051 |
| Albania | Alcohol use | 1.8 | 1.18 | 2.43 | <0.001 |
| Albania | All cause | -1.57 | -1.87 | -1.28 | <0.001 |
| Albania | Smoking | -1.39 | -1.7 | -1.09 | <0.001 |
| Algeria | Alcohol use | 0.68 | 0.38 | 0.98 | <0.001 |
| Algeria | All cause | -2.03 | -2.26 | -1.79 | <0.001 |
| Algeria | Smoking | -2.19 | -2.44 | -1.94 | <0.001 |
| American Samoa | Alcohol use | -2.75 | -3.35 | -2.16 | <0.001 |
| American Samoa | All cause | -2.42 | -2.65 | -2.19 | <0.001 |
| American Samoa | Smoking | -2.77 | -3.01 | -2.52 | <0.001 |
| Andorra | Alcohol use | -2.35 | -2.45 | -2.25 | <0.001 |
| Andorra | All cause | -2.08 | -2.14 | -2.01 | <0.001 |
| Andorra | Smoking | -2.39 | -2.46 | -2.32 | <0.001 |
| Angola | Alcohol use | 3.27 | 2.84 | 3.71 | <0.001 |
| Angola | All cause | -0.6 | -0.68 | -0.53 | <0.001 |
| Angola | Smoking | -0.71 | -0.84 | -0.57 | <0.001 |
| Antigua | Alcohol use | 2.24 | 1.78 | 2.71 | <0.001 |
| Antigua | All cause | -0.11 | -0.43 | 0.21 | 0.472 |
| Antigua | Smoking | -0.17 | -0.49 | 0.16 | 0.299 |
| Argentina | Alcohol use | -2.78 | -2.98 | -2.59 | <0.001 |
| Argentina | All cause | -2.14 | -2.37 | -1.9 | <0.001 |
| Argentina | Smoking | -2.47 | -2.72 | -2.22 | <0.001 |
| Armenia | Alcohol use | -0.71 | -1 | -0.41 | <0.001 |
| Armenia | All cause | -2.2 | -2.37 | -2.02 | <0.001 |
| Armenia | Smoking | -2.32 | -2.49 | -2.15 | <0.001 |
| Australia | Alcohol use | -3.04 | -3.17 | -2.9 | <0.001 |
| Australia | All cause | -2.86 | -2.99 | -2.73 | <0.001 |
| Australia | Smoking | -4.09 | -4.23 | -3.95 | <0.001 |
| Austria | Alcohol use | -2.99 | -3.09 | -2.89 | <0.001 |
| Austria | All cause | -2.76 | -2.85 | -2.66 | <0.001 |
| Austria | Smoking | -2.86 | -2.96 | -2.76 | <0.001 |
| Azerbaijan | Alcohol use | -1.57 | -1.99 | -1.15 | <0.001 |
| Azerbaijan | All cause | -1.47 | -1.7 | -1.25 | <0.001 |
| Azerbaijan | Smoking | -1.45 | -1.66 | -1.23 | <0.001 |
| Bahamas | Alcohol use | -1.61 | -1.8 | -1.41 | <0.001 |
| Bahamas | All cause | -0.33 | -0.44 | -0.22 | <0.001 |
| Bahamas | Smoking | -0.49 | -0.62 | -0.37 | <0.001 |
| Bahrain | Alcohol use | -7.05 | -7.63 | -6.47 | <0.001 |
| Bahrain | All cause | -4.19 | -4.67 | -3.71 | <0.001 |
| Bahrain | Smoking | -4.27 | -4.75 | -3.79 | <0.001 |
| Bangladesh | Alcohol use | 2.31 | 1.93 | 2.69 | <0.001 |
| Bangladesh | All cause | -2.04 | -2.25 | -1.84 | <0.001 |
| Bangladesh | Smoking | -2.33 | -2.54 | -2.11 | <0.001 |
| Barbados | Alcohol use | 0.58 | 0.42 | 0.74 | <0.001 |
| Barbados | All cause | -0.13 | -0.3 | 0.03 | 0.112 |
| Barbados | Smoking | -0.57 | -0.79 | -0.36 | <0.001 |
| Belarus | Alcohol use | -2.94 | -3.48 | -2.39 | <0.001 |
| Belarus | All cause | -3.17 | -3.67 | -2.67 | <0.001 |
| Belarus | Smoking | -3.14 | -3.64 | -2.64 | <0.001 |
| Belgium | Alcohol use | -3.57 | -3.69 | -3.46 | <0.001 |
| Belgium | All cause | -3.42 | -3.54 | -3.3 | <0.001 |
| Belgium | Smoking | -3.74 | -3.86 | -3.62 | <0.001 |
| Belize | Alcohol use | 1.35 | 0.81 | 1.89 | <0.001 |
| Belize | All cause | 0.88 | 0.33 | 1.43 | 0.003 |
| Belize | Smoking | 0.96 | 0.42 | 1.52 | 0.001 |
| Benin | Alcohol use | 1.33 | 1.12 | 1.54 | <0.001 |
| Benin | All cause | 0.35 | 0.21 | 0.48 | <0.001 |
| Benin | Smoking | -0.45 | -0.57 | -0.32 | <0.001 |
| Bermuda | Alcohol use | -1.58 | -1.76 | -1.4 | <0.001 |
| Bermuda | All cause | -1.27 | -1.42 | -1.12 | <0.001 |
| Bermuda | Smoking | -1.29 | -1.43 | -1.14 | <0.001 |
| Bhutan | Alcohol use | -5.12 | -5.6 | -4.63 | <0.001 |
| Bhutan | All cause | -0.88 | -0.94 | -0.82 | <0.001 |
| Bhutan | Smoking | -1.06 | -1.16 | -0.97 | <0.001 |
| Bolivia | Alcohol use | -0.55 | -0.87 | -0.24 | 0.001 |
| Bolivia | All cause | -0.98 | -1.17 | -0.79 | <0.001 |
| Bolivia | Smoking | -1.3 | -1.62 | -0.97 | <0.001 |
| Bosnia and Herzegovina | Alcohol use | -1.14 | -1.47 | -0.82 | <0.001 |
| Bosnia and Herzegovina | All cause | -2.05 | -2.32 | -1.78 | <0.001 |
| Bosnia and Herzegovina | Smoking | -1.92 | -2.15 | -1.68 | <0.001 |
| Botswana | Alcohol use | -0.91 | -1.6 | -0.22 | 0.012 |
| Botswana | All cause | -0.97 | -1.54 | -0.39 | 0.002 |
| Botswana | Smoking | -1.15 | -1.72 | -0.58 | <0.001 |
| Brazil | Alcohol use | -0.53 | -0.79 | -0.27 | <0.001 |
| Brazil | All cause | -0.96 | -1.04 | -0.88 | <0.001 |
| Brazil | Smoking | -2.11 | -2.26 | -1.96 | <0.001 |
| Brunei | Alcohol use | -6.87 | -8.34 | -5.37 | <0.001 |
| Brunei | All cause | -4.65 | -4.96 | -4.34 | <0.001 |
| Brunei | Smoking | -4.89 | -5.2 | -4.58 | <0.001 |
| Bulgaria | Alcohol use | 1.52 | 1.11 | 1.93 | <0.001 |
| Bulgaria | All cause | 1.32 | 0.93 | 1.71 | <0.001 |
| Bulgaria | Smoking | 1.21 | 0.81 | 1.62 | <0.001 |
| Burkina Faso | Alcohol use | 1.43 | 1.33 | 1.53 | <0.001 |
| Burkina Faso | All cause | 0.73 | 0.58 | 0.88 | <0.001 |
| Burkina Faso | Smoking | 0.32 | 0.18 | 0.45 | <0.001 |
| Burundi | Alcohol use | -3 | -3.22 | -2.78 | <0.001 |
| Burundi | All cause | -2.1 | -2.27 | -1.93 | <0.001 |
| Burundi | Smoking | -2.68 | -3.02 | -2.33 | <0.001 |
| Cambodia | Alcohol use | 5.02 | 4.74 | 5.3 | <0.001 |
| Cambodia | All cause | -0.58 | -0.8 | -0.36 | <0.001 |
| Cambodia | Smoking | -0.6 | -0.82 | -0.39 | <0.001 |
| Cameroon | Alcohol use | 1.59 | 1.35 | 1.82 | <0.001 |
| Cameroon | All cause | 0.95 | 0.73 | 1.16 | <0.001 |
| Cameroon | Smoking | 0.69 | 0.44 | 0.93 | <0.001 |
| Canada | Alcohol use | -2.71 | -2.88 | -2.53 | <0.001 |
| Canada | All cause | -2.83 | -2.99 | -2.66 | <0.001 |
| Canada | Smoking | -3.44 | -3.62 | -3.26 | <0.001 |
| Cape Verde | Alcohol use | -1.02 | -1.53 | -0.52 | <0.001 |
| Cape Verde | All cause | -1.4 | -1.95 | -0.84 | <0.001 |
| Cape Verde | Smoking | -2.6 | -3.23 | -1.97 | <0.001 |
| Central African Republic | Alcohol use | -2.02 | -2.24 | -1.8 | <0.001 |
| Central African Republic | All cause | -1.08 | -1.11 | -1.05 | <0.001 |
| Central African Republic | Smoking | -1.71 | -1.81 | -1.62 | <0.001 |
| Chad | Alcohol use | 4.14 | 3.81 | 4.47 | <0.001 |
| Chad | All cause | 1.39 | 1.18 | 1.61 | <0.001 |
| Chad | Smoking | 0.84 | 0.57 | 1.11 | <0.001 |
| Chile | Alcohol use | -2.24 | -2.46 | -2.02 | <0.001 |
| Chile | All cause | -2.3 | -2.44 | -2.15 | <0.001 |
| Chile | Smoking | -3.07 | -3.17 | -2.98 | <0.001 |
| China | Alcohol use | -0.34 | -0.55 | -0.12 | 0.004 |
| China | All cause | -0.95 | -1.06 | -0.85 | <0.001 |
| China | Smoking | -0.84 | -0.94 | -0.73 | <0.001 |
| Colombia | Alcohol use | -4.12 | -4.45 | -3.79 | <0.001 |
| Colombia | All cause | -3.58 | -3.83 | -3.34 | <0.001 |
| Colombia | Smoking | -4.77 | -5.03 | -4.51 | <0.001 |
| Comoros | Alcohol use | 1.23 | 1.07 | 1.38 | <0.001 |
| Comoros | All cause | -0.98 | -1.16 | -0.8 | <0.001 |
| Comoros | Smoking | -1.33 | -1.51 | -1.14 | <0.001 |
| Cook Islands | Alcohol use | 4.22 | 3.25 | 5.2 | <0.001 |
| Cook Islands | All cause | -1.76 | -2.02 | -1.49 | <0.001 |
| Cook Islands | Smoking | -2.1 | -2.37 | -1.83 | <0.001 |
| Costa Rica | Alcohol use | -2.79 | -3.12 | -2.47 | <0.001 |
| Costa Rica | All cause | -1.73 | -1.92 | -1.53 | <0.001 |
| Costa Rica | Smoking | -2.36 | -2.53 | -2.19 | <0.001 |
| Croatia | Alcohol use | -2.76 | -2.92 | -2.59 | <0.001 |
| Croatia | All cause | -2.51 | -2.65 | -2.37 | <0.001 |
| Croatia | Smoking | -2.62 | -2.75 | -2.49 | <0.001 |
| Cuba | Alcohol use | 1.71 | 1.57 | 1.86 | <0.001 |
| Cuba | All cause | 0.69 | 0.6 | 0.79 | <0.001 |
| Cuba | Smoking | 0.47 | 0.36 | 0.58 | <0.001 |
| Cyprus | Alcohol use | -1.63 | -1.91 | -1.35 | <0.001 |
| Cyprus | All cause | -1.84 | -2.04 | -1.65 | <0.001 |
| Cyprus | Smoking | -1.87 | -2.09 | -1.66 | <0.001 |
| Czech Republic | Alcohol use | -2.45 | -2.53 | -2.36 | <0.001 |
| Czech Republic | All cause | -2.29 | -2.36 | -2.21 | <0.001 |
| Czech Republic | Smoking | -2.51 | -2.59 | -2.44 | <0.001 |
| Côte d'Ivoire | Alcohol use | 0.84 | 0.67 | 1.01 | <0.001 |
| Côte d'Ivoire | All cause | 0.14 | -0.01 | 0.3 | 0.072 |
| Côte d'Ivoire | Smoking | 0.28 | 0.04 | 0.52 | 0.023 |
| Democratic Republic of the Congo | Alcohol use | -1.69 | -2.67 | -0.7 | 0.002 |
| Democratic Republic of the Congo | All cause | -1.01 | -1.11 | -0.92 | <0.001 |
| Democratic Republic of the Congo | Smoking | -1.12 | -1.34 | -0.89 | <0.001 |
| Denmark | Alcohol use | -3.44 | -3.57 | -3.31 | <0.001 |
| Denmark | All cause | -2.95 | -3.04 | -2.86 | <0.001 |
| Denmark | Smoking | -3.38 | -3.48 | -3.28 | <0.001 |
| Djibouti | Alcohol use | -3.74 | -3.89 | -3.59 | <0.001 |
| Djibouti | All cause | -0.62 | -0.74 | -0.5 | <0.001 |
| Djibouti | Smoking | -0.3 | -0.41 | -0.18 | <0.001 |
| Dominica | Alcohol use | 0.59 | 0.49 | 0.7 | <0.001 |
| Dominica | All cause | 0.44 | 0.37 | 0.52 | <0.001 |
| Dominica | Smoking | 0.31 | 0.19 | 0.44 | <0.001 |
| Dominican Republic | Alcohol use | 1.39 | 1.19 | 1.59 | <0.001 |
| Dominican Republic | All cause | 1.22 | 0.94 | 1.51 | <0.001 |
| Dominican Republic | Smoking | 1.01 | 0.77 | 1.24 | <0.001 |
| Ecuador | Alcohol use | 0.54 | 0.19 | 0.9 | 0.004 |
| Ecuador | All cause | -0.59 | -0.85 | -0.34 | <0.001 |
| Ecuador | Smoking | -1.45 | -1.72 | -1.17 | <0.001 |
| Egypt | Alcohol use | 0.94 | 0.73 | 1.15 | <0.001 |
| Egypt | All cause | 0.49 | 0.27 | 0.71 | <0.001 |
| Egypt | Smoking | 0.71 | 0.43 | 0.99 | <0.001 |
| El Salvador | Alcohol use | -0.04 | -0.38 | 0.3 | 0.819 |
| El Salvador | All cause | -0.19 | -0.44 | 0.06 | 0.127 |
| El Salvador | Smoking | 0.06 | -0.21 | 0.33 | 0.676 |
| Equatorial Guinea | Alcohol use | 0.85 | 0.62 | 1.07 | <0.001 |
| Equatorial Guinea | All cause | -1.81 | -2.23 | -1.39 | <0.001 |
| Equatorial Guinea | Smoking | -1.85 | -2.38 | -1.32 | <0.001 |
| Eritrea | Alcohol use | -1.32 | -1.76 | -0.87 | <0.001 |
| Eritrea | All cause | -0.82 | -0.97 | -0.67 | <0.001 |
| Eritrea | Smoking | -1.47 | -1.65 | -1.3 | <0.001 |
| Estonia | Alcohol use | -1.99 | -2.32 | -1.67 | <0.001 |
| Estonia | All cause | -2.84 | -3.12 | -2.57 | <0.001 |
| Estonia | Smoking | -2.93 | -3.22 | -2.63 | <0.001 |
| Ethiopia | Alcohol use | -1.37 | -1.79 | -0.96 | <0.001 |
| Ethiopia | All cause | -2.66 | -2.8 | -2.53 | <0.001 |
| Ethiopia | Smoking | -2.75 | -3.12 | -2.38 | <0.001 |
| Fiji | Alcohol use | 0.89 | 0.53 | 1.25 | <0.001 |
| Fiji | All cause | -0.19 | -0.35 | -0.02 | 0.028 |
| Fiji | Smoking | -0.79 | -1 | -0.59 | <0.001 |
| Finland | Alcohol use | -1.96 | -2.12 | -1.79 | <0.001 |
| Finland | All cause | -2.07 | -2.25 | -1.88 | <0.001 |
| Finland | Smoking | -2.48 | -2.65 | -2.3 | <0.001 |
| France | Alcohol use | -3.96 | -4.35 | -3.57 | <0.001 |
| France | All cause | -3.64 | -4.02 | -3.27 | <0.001 |
| France | Smoking | -4.02 | -4.4 | -3.63 | <0.001 |
| Gabon | Alcohol use | -1.4 | -1.55 | -1.25 | <0.001 |
| Gabon | All cause | -1.1 | -1.16 | -1.05 | <0.001 |
| Gabon | Smoking | -0.88 | -0.94 | -0.83 | <0.001 |
| Gambia | Alcohol use | 1.64 | 1.25 | 2.03 | <0.001 |
| Gambia | All cause | 0.01 | -0.15 | 0.18 | 0.859 |
| Gambia | Smoking | -0.81 | -0.99 | -0.63 | <0.001 |
| Georgia | Alcohol use | 1.12 | 0.49 | 1.76 | 0.001 |
| Georgia | All cause | -0.44 | -0.83 | -0.06 | 0.026 |
| Georgia | Smoking | -0.16 | -0.59 | 0.26 | 0.439 |
| Germany | Alcohol use | -2.09 | -2.16 | -2.02 | <0.001 |
| Germany | All cause | -1.88 | -1.94 | -1.83 | <0.001 |
| Germany | Smoking | -2.16 | -2.21 | -2.11 | <0.001 |
| Ghana | Alcohol use | 1.85 | 1.6 | 2.1 | <0.001 |
| Ghana | All cause | 1.31 | 1.07 | 1.56 | <0.001 |
| Ghana | Smoking | 1.45 | 1.16 | 1.75 | <0.001 |
| Greece | Alcohol use | -1.54 | -1.64 | -1.44 | <0.001 |
| Greece | All cause | -1.21 | -1.28 | -1.14 | <0.001 |
| Greece | Smoking | -1.27 | -1.34 | -1.19 | <0.001 |
| Greenland | Alcohol use | -1.07 | -1.34 | -0.79 | <0.001 |
| Greenland | All cause | -1.29 | -1.48 | -1.11 | <0.001 |
| Greenland | Smoking | -1.57 | -1.75 | -1.39 | <0.001 |
| Grenada | Alcohol use | -0.5 | -0.92 | -0.07 | 0.024 |
| Grenada | All cause | -0.77 | -1.17 | -0.37 | 0.001 |
| Grenada | Smoking | -1.02 | -1.45 | -0.58 | <0.001 |
| Guam | Alcohol use | -0.88 | -1.34 | -0.43 | <0.001 |
| Guam | All cause | -3.15 | -3.5 | -2.8 | <0.001 |
| Guam | Smoking | -3.34 | -3.68 | -2.99 | <0.001 |
| Guatemala | Alcohol use | -2.53 | -2.82 | -2.23 | <0.001 |
| Guatemala | All cause | -2.32 | -2.59 | -2.06 | <0.001 |
| Guatemala | Smoking | -2.81 | -3.03 | -2.59 | <0.001 |
| Guinea | Alcohol use | 2.54 | 2.44 | 2.64 | <0.001 |
| Guinea | All cause | 1.72 | 1.51 | 1.92 | <0.001 |
| Guinea | Smoking | 1.97 | 1.74 | 2.2 | <0.001 |
| Guinea-Bissau | Alcohol use | 0.04 | -0.17 | 0.26 | 0.682 |
| Guinea-Bissau | All cause | 0.06 | -0.13 | 0.24 | 0.531 |
| Guinea-Bissau | Smoking | 0.89 | 0.55 | 1.23 | <0.001 |
| Guyana | Alcohol use | -0.52 | -0.61 | -0.43 | <0.001 |
| Guyana | All cause | -0.12 | -0.22 | -0.02 | 0.015 |
| Guyana | Smoking | -0.39 | -0.48 | -0.29 | <0.001 |
| Haiti | Alcohol use | -0.52 | -0.66 | -0.38 | <0.001 |
| Haiti | All cause | -0.55 | -0.7 | -0.41 | <0.001 |
| Haiti | Smoking | -1.61 | -1.82 | -1.41 | <0.001 |
| Honduras | Alcohol use | 0.95 | 0.82 | 1.09 | <0.001 |
| Honduras | All cause | 0.89 | 0.68 | 1.1 | <0.001 |
| Honduras | Smoking | 0.74 | 0.51 | 0.97 | <0.001 |
| Hungary | Alcohol use | -2.15 | -2.39 | -1.92 | <0.001 |
| Hungary | All cause | -1.79 | -2.04 | -1.54 | <0.001 |
| Hungary | Smoking | -1.97 | -2.25 | -1.7 | <0.001 |
| Iceland | Alcohol use | -0.87 | -1.01 | -0.72 | <0.001 |
| Iceland | All cause | -2.31 | -2.53 | -2.09 | <0.001 |
| Iceland | Smoking | -2.79 | -3.01 | -2.57 | <0.001 |
| India | Alcohol use | 0.54 | 0.22 | 0.86 | 0.002 |
| India | All cause | -1.1 | -1.22 | -0.98 | <0.001 |
| India | Smoking | -1.62 | -1.74 | -1.5 | <0.001 |
| Indonesia | Alcohol use | -0.35 | -0.52 | -0.18 | <0.001 |
| Indonesia | All cause | -0.04 | -0.12 | 0.04 | 0.296 |
| Indonesia | Smoking | 0.22 | 0.12 | 0.32 | <0.001 |
| Iran | Alcohol use | 3.7 | 3.14 | 4.26 | <0.001 |
| Iran | All cause | -1.33 | -1.53 | -1.14 | <0.001 |
| Iran | Smoking | -1.25 | -1.39 | -1.11 | <0.001 |
| Iraq | Alcohol use | -3.33 | -3.84 | -2.83 | <0.001 |
| Iraq | All cause | -0.53 | -0.63 | -0.43 | <0.001 |
| Iraq | Smoking | -0.74 | -0.84 | -0.63 | <0.001 |
| Ireland | Alcohol use | -2.29 | -2.53 | -2.06 | <0.001 |
| Ireland | All cause | -2.22 | -2.36 | -2.09 | <0.001 |
| Ireland | Smoking | -2.8 | -2.97 | -2.63 | <0.001 |
| Israel | Alcohol use | 0.74 | 0.57 | 0.9 | <0.001 |
| Israel | All cause | -1.31 | -1.58 | -1.03 | <0.001 |
| Israel | Smoking | -1.66 | -1.95 | -1.38 | <0.001 |
| Italy | Alcohol use | -3.81 | -3.96 | -3.66 | <0.001 |
| Italy | All cause | -3.14 | -3.27 | -3.01 | <0.001 |
| Italy | Smoking | -3.56 | -3.7 | -3.43 | <0.001 |
| Jamaica | Alcohol use | 1.55 | 1.07 | 2.04 | <0.001 |
| Jamaica | All cause | 0.78 | 0.33 | 1.23 | 0.001 |
| Jamaica | Smoking | 0.41 | -0.1 | 0.93 | 0.113 |
| Japan | Alcohol use | -2.78 | -2.92 | -2.64 | <0.001 |
| Japan | All cause | -2.51 | -2.59 | -2.43 | <0.001 |
| Japan | Smoking | -2.97 | -3.07 | -2.88 | <0.001 |
| Jordan | Alcohol use | -2.47 | -3.4 | -1.53 | <0.001 |
| Jordan | All cause | -4.31 | -4.95 | -3.67 | <0.001 |
| Jordan | Smoking | -4.3 | -4.92 | -3.66 | <0.001 |
| Kazakhstan | Alcohol use | -4.14 | -4.46 | -3.82 | <0.001 |
| Kazakhstan | All cause | -3.34 | -3.56 | -3.13 | <0.001 |
| Kazakhstan | Smoking | -3.39 | -3.62 | -3.16 | <0.001 |
| Kenya | Alcohol use | 0.34 | 0.12 | 0.55 | 0.003 |
| Kenya | All cause | 0.78 | 0.49 | 1.07 | <0.001 |
| Kenya | Smoking | -0.06 | -0.37 | 0.25 | 0.687 |
| Kiribati | Alcohol use | -1.15 | -1.32 | -0.98 | <0.001 |
| Kiribati | All cause | -0.38 | -0.43 | -0.33 | <0.001 |
| Kiribati | Smoking | -0.35 | -0.4 | -0.29 | <0.001 |
| Kuwait | Alcohol use | 0.68 | 0.56 | 0.8 | <0.001 |
| Kuwait | All cause | -1.45 | -1.82 | -1.07 | <0.001 |
| Kuwait | Smoking | -1.44 | -1.8 | -1.07 | <0.001 |
| Kyrgyzstan | Alcohol use | -3.08 | -3.41 | -2.75 | <0.001 |
| Kyrgyzstan | All cause | -3.67 | -4.08 | -3.25 | <0.001 |
| Kyrgyzstan | Smoking | -3.66 | -4.09 | -3.23 | <0.001 |
| Laos | Alcohol use | -0.91 | -1.38 | -0.43 | 0.001 |
| Laos | All cause | -2.16 | -2.33 | -1.98 | <0.001 |
| Laos | Smoking | -2.06 | -2.26 | -1.87 | <0.001 |
| Latvia | Alcohol use | -1.12 | -1.52 | -0.72 | <0.001 |
| Latvia | All cause | -1.85 | -2.2 | -1.5 | <0.001 |
| Latvia | Smoking | -1.95 | -2.31 | -1.59 | <0.001 |
| Lebanon | Alcohol use | -2.45 | -2.71 | -2.19 | <0.001 |
| Lebanon | All cause | -0.95 | -1.15 | -0.74 | <0.001 |
| Lebanon | Smoking | -0.76 | -0.96 | -0.57 | <0.001 |
| Lesotho | Alcohol use | 1.81 | 1.5 | 2.11 | <0.001 |
| Lesotho | All cause | 1.42 | 1.13 | 1.71 | <0.001 |
| Lesotho | Smoking | 1.36 | 1.09 | 1.62 | <0.001 |
| Liberia | Alcohol use | -0.44 | -0.7 | -0.19 | 0.002 |
| Liberia | All cause | -0.04 | -0.24 | 0.16 | 0.683 |
| Liberia | Smoking | -0.29 | -0.5 | -0.09 | 0.007 |
| Libya | Alcohol use | 2.11 | 1.08 | 3.15 | <0.001 |
| Libya | All cause | -0.54 | -0.71 | -0.36 | <0.001 |
| Libya | Smoking | -0.59 | -0.78 | -0.4 | <0.001 |
| Lithuania | Alcohol use | -0.79 | -1.2 | -0.37 | 0.001 |
| Lithuania | All cause | -1.56 | -1.91 | -1.22 | <0.001 |
| Lithuania | Smoking | -1.66 | -2.01 | -1.3 | <0.001 |
| Luxembourg | Alcohol use | -3.72 | -3.82 | -3.62 | <0.001 |
| Luxembourg | All cause | -3.44 | -3.52 | -3.36 | <0.001 |
| Luxembourg | Smoking | -3.82 | -3.9 | -3.74 | <0.001 |
| Macedonia | Alcohol use | -1.02 | -1.2 | -0.85 | <0.001 |
| Macedonia | All cause | -0.31 | -0.59 | -0.04 | 0.026 |
| Macedonia | Smoking | -0.37 | -0.65 | -0.09 | 0.012 |
| Madagascar | Alcohol use | -1.56 | -1.87 | -1.25 | <0.001 |
| Madagascar | All cause | -1.04 | -1.16 | -0.92 | <0.001 |
| Madagascar | Smoking | -2.23 | -2.4 | -2.05 | <0.001 |
| Malawi | Alcohol use | 0.85 | 0.71 | 0.99 | <0.001 |
| Malawi | All cause | -0.67 | -0.86 | -0.48 | <0.001 |
| Malawi | Smoking | -0.64 | -0.87 | -0.41 | <0.001 |
| Malaysia | Alcohol use | -3.07 | -3.66 | -2.49 | <0.001 |
| Malaysia | All cause | -1.55 | -1.88 | -1.23 | <0.001 |
| Malaysia | Smoking | -1.88 | -2.2 | -1.56 | <0.001 |
| Maldives | Alcohol use | -1.26 | -2.76 | 0.26 | 0.101 |
| Maldives | All cause | -3.25 | -3.49 | -3 | <0.001 |
| Maldives | Smoking | -3.22 | -3.47 | -2.96 | <0.001 |
| Mali | Alcohol use | 0.23 | 0.12 | 0.35 | <0.001 |
| Mali | All cause | -0.29 | -0.41 | -0.18 | <0.001 |
| Mali | Smoking | 0.59 | 0.45 | 0.73 | <0.001 |
| Malta | Alcohol use | -2.26 | -2.45 | -2.06 | <0.001 |
| Malta | All cause | -2.61 | -2.72 | -2.5 | <0.001 |
| Malta | Smoking | -2.93 | -3.07 | -2.79 | <0.001 |
| Marshall Islands | Alcohol use | -0.02 | -0.14 | 0.1 | 0.735 |
| Marshall Islands | All cause | -0.28 | -0.42 | -0.14 | <0.001 |
| Marshall Islands | Smoking | -0.3 | -0.47 | -0.13 | 0.001 |
| Mauritania | Alcohol use | -0.27 | -0.32 | -0.23 | <0.001 |
| Mauritania | All cause | -0.34 | -0.58 | -0.09 | 0.009 |
| Mauritania | Smoking | -0.81 | -1.08 | -0.55 | <0.001 |
| Mauritius | Alcohol use | -2.32 | -2.58 | -2.06 | <0.001 |
| Mauritius | All cause | -2.39 | -2.58 | -2.2 | <0.001 |
| Mauritius | Smoking | -2.19 | -2.43 | -1.95 | <0.001 |
| Mexico | Alcohol use | -2.41 | -2.6 | -2.22 | <0.001 |
| Mexico | All cause | -2.73 | -2.9 | -2.57 | <0.001 |
| Mexico | Smoking | -3.76 | -3.95 | -3.56 | <0.001 |
| Micronesia | Alcohol use | -2.5 | -2.79 | -2.2 | <0.001 |
| Micronesia | All cause | -0.97 | -1.08 | -0.86 | <0.001 |
| Micronesia | Smoking | -1.1 | -1.22 | -0.98 | <0.001 |
| Monaco | Alcohol use | -1.77 | -2.02 | -1.52 | <0.001 |
| Monaco | All cause | -1.65 | -1.83 | -1.47 | <0.001 |
| Monaco | Smoking | -1.99 | -2.17 | -1.8 | <0.001 |
| Mongolia | Alcohol use | 4.99 | 4.47 | 5.51 | <0.001 |
| Mongolia | All cause | 1.51 | 1.15 | 1.87 | <0.001 |
| Mongolia | Smoking | 2.21 | 1.82 | 2.59 | <0.001 |
| Montenegro | Alcohol use | -0.79 | -0.94 | -0.65 | <0.001 |
| Montenegro | All cause | -0.27 | -0.48 | -0.05 | 0.017 |
| Montenegro | Smoking | -0.35 | -0.59 | -0.1 | 0.008 |
| Morocco | Alcohol use | -2.02 | -2.15 | -1.88 | <0.001 |
| Morocco | All cause | -0.42 | -0.66 | -0.19 | 0.001 |
| Morocco | Smoking | -0.87 | -1.14 | -0.61 | <0.001 |
| Mozambique | Alcohol use | 4.23 | 4.06 | 4.39 | <0.001 |
| Mozambique | All cause | 0.9 | 0.75 | 1.05 | <0.001 |
| Mozambique | Smoking | 1 | 0.82 | 1.18 | <0.001 |
| Myanmar | Alcohol use | 4.53 | 4.22 | 4.85 | <0.001 |
| Myanmar | All cause | -2.11 | -2.19 | -2.03 | <0.001 |
| Myanmar | Smoking | -2.72 | -2.82 | -2.63 | <0.001 |
| Namibia | Alcohol use | 1.71 | 1.46 | 1.97 | <0.001 |
| Namibia | All cause | 0.16 | -0.1 | 0.42 | 0.219 |
| Namibia | Smoking | -0.47 | -0.73 | -0.2 | 0.001 |
| Nauru | Alcohol use | -0.28 | -0.64 | 0.08 | 0.122 |
| Nauru | All cause | -0.64 | -0.96 | -0.33 | <0.001 |
| Nauru | Smoking | -0.93 | -1.33 | -0.54 | <0.001 |
| Nepal | Alcohol use | 6.66 | 6.03 | 7.29 | <0.001 |
| Nepal | All cause | -0.55 | -0.87 | -0.23 | 0.001 |
| Nepal | Smoking | -1.27 | -1.53 | -1.01 | <0.001 |
| Netherlands | Alcohol use | -2.7 | -2.95 | -2.45 | <0.001 |
| Netherlands | All cause | -2.34 | -2.58 | -2.1 | <0.001 |
| Netherlands | Smoking | -2.89 | -3.14 | -2.64 | <0.001 |
| New Zealand | Alcohol use | -2.72 | -2.9 | -2.54 | <0.001 |
| New Zealand | All cause | -2.4 | -2.52 | -2.28 | <0.001 |
| New Zealand | Smoking | -3.19 | -3.31 | -3.07 | <0.001 |
| Nicaragua | Alcohol use | -0.06 | -0.17 | 0.06 | 0.333 |
| Nicaragua | All cause | -0.99 | -1.21 | -0.77 | <0.001 |
| Nicaragua | Smoking | -1.21 | -1.4 | -1.03 | <0.001 |
| Niger | Alcohol use | 0.94 | 0.47 | 1.4 | <0.001 |
| Niger | All cause | 0.4 | 0.23 | 0.57 | <0.001 |
| Niger | Smoking | 0.9 | 0.74 | 1.07 | <0.001 |
| Nigeria | Alcohol use | 0.13 | -0.17 | 0.43 | 0.375 |
| Nigeria | All cause | -0.26 | -0.46 | -0.07 | 0.011 |
| Nigeria | Smoking | -1.06 | -1.23 | -0.88 | <0.001 |
| Niue | Alcohol use | -1.5 | -1.8 | -1.21 | <0.001 |
| Niue | All cause | -1.02 | -1.15 | -0.89 | <0.001 |
| Niue | Smoking | -1.22 | -1.38 | -1.07 | <0.001 |
| North Korea | Alcohol use | -0.6 | -0.67 | -0.52 | <0.001 |
| North Korea | All cause | -0.42 | -0.48 | -0.36 | <0.001 |
| North Korea | Smoking | -0.35 | -0.42 | -0.27 | <0.001 |
| Northern Mariana Islands | Alcohol use | -3.14 | -3.77 | -2.5 | <0.001 |
| Northern Mariana Islands | All cause | -3.09 | -3.64 | -2.53 | <0.001 |
| Northern Mariana Islands | Smoking | -3.91 | -4.47 | -3.35 | <0.001 |
| Norway | Alcohol use | -1.49 | -1.69 | -1.29 | <0.001 |
| Norway | All cause | -2.16 | -2.26 | -2.06 | <0.001 |
| Norway | Smoking | -3.68 | -3.88 | -3.47 | <0.001 |
| Oman | Alcohol use | -0.5 | -1.54 | 0.56 | 0.343 |
| Oman | All cause | -1.08 | -1.31 | -0.84 | <0.001 |
| Oman | Smoking | -1.34 | -1.54 | -1.13 | <0.001 |
| Pakistan | Alcohol use | 1.02 | 0.8 | 1.23 | <0.001 |
| Pakistan | All cause | -0.4 | -0.66 | -0.13 | 0.005 |
| Pakistan | Smoking | -1.07 | -1.38 | -0.75 | <0.001 |
| Palau | Alcohol use | -0.02 | -0.14 | 0.11 | 0.801 |
| Palau | All cause | -0.71 | -0.79 | -0.62 | <0.001 |
| Palau | Smoking | -1.17 | -1.21 | -1.13 | <0.001 |
| Palestine | Alcohol use | -0.61 | -1 | -0.22 | 0.003 |
| Palestine | All cause | -1.73 | -1.88 | -1.58 | <0.001 |
| Palestine | Smoking | -1.77 | -1.92 | -1.62 | <0.001 |
| Panama | Alcohol use | -2.19 | -2.48 | -1.91 | <0.001 |
| Panama | All cause | -2.56 | -2.75 | -2.37 | <0.001 |
| Panama | Smoking | -3.39 | -3.58 | -3.19 | <0.001 |
| Papua New Guinea | Alcohol use | -0.99 | -1.46 | -0.52 | <0.001 |
| Papua New Guinea | All cause | -0.26 | -0.28 | -0.23 | <0.001 |
| Papua New Guinea | Smoking | -0.76 | -0.82 | -0.7 | <0.001 |
| Paraguay | Alcohol use | 0.21 | 0.02 | 0.39 | 0.033 |
| Paraguay | All cause | 0.74 | 0.62 | 0.86 | <0.001 |
| Paraguay | Smoking | 0.26 | 0.11 | 0.4 | 0.001 |
| Peru | Alcohol use | -2.65 | -3.2 | -2.1 | <0.001 |
| Peru | All cause | -2.96 | -3.32 | -2.61 | <0.001 |
| Peru | Smoking | -3.67 | -3.97 | -3.37 | <0.001 |
| Philippines | Alcohol use | -0.64 | -0.85 | -0.43 | <0.001 |
| Philippines | All cause | -1.11 | -1.3 | -0.91 | <0.001 |
| Philippines | Smoking | -1.42 | -1.6 | -1.23 | <0.001 |
| Poland | Alcohol use | -1.79 | -1.9 | -1.68 | <0.001 |
| Poland | All cause | -1.99 | -2.11 | -1.87 | <0.001 |
| Poland | Smoking | -2.25 | -2.37 | -2.13 | <0.001 |
| Portugal | Alcohol use | -2.45 | -2.69 | -2.22 | <0.001 |
| Portugal | All cause | -2.28 | -2.48 | -2.07 | <0.001 |
| Portugal | Smoking | -2.56 | -2.79 | -2.32 | <0.001 |
| Puerto Rico | Alcohol use | -4.39 | -4.8 | -3.99 | <0.001 |
| Puerto Rico | All cause | -4.17 | -4.48 | -3.85 | <0.001 |
| Puerto Rico | Smoking | -4.48 | -4.81 | -4.16 | <0.001 |
| Qatar | Alcohol use | -2.95 | -3.35 | -2.55 | <0.001 |
| Qatar | All cause | -1.61 | -2.04 | -1.18 | <0.001 |
| Qatar | Smoking | -1.91 | -2.34 | -1.48 | <0.001 |
| Republic of Congo | Alcohol use | -0.66 | -1.38 | 0.07 | 0.075 |
| Republic of Congo | All cause | -1.69 | -1.92 | -1.47 | <0.001 |
| Republic of Congo | Smoking | -1.49 | -1.77 | -1.22 | <0.001 |
| Republic of Moldova | Alcohol use | -1.2 | -1.57 | -0.83 | <0.001 |
| Republic of Moldova | All cause | -0.65 | -1.01 | -0.29 | 0.001 |
| Republic of Moldova | Smoking | -0.59 | -0.95 | -0.22 | 0.003 |
| Romania | Alcohol use | -0.05 | -0.26 | 0.16 | 0.634 |
| Romania | All cause | -0.22 | -0.43 | 0 | 0.047 |
| Romania | Smoking | -0.36 | -0.58 | -0.14 | 0.002 |
| Russia | Alcohol use | -2.83 | -3.32 | -2.34 | <0.001 |
| Russia | All cause | -2.82 | -3.2 | -2.43 | <0.001 |
| Russia | Smoking | -2.83 | -3.23 | -2.42 | <0.001 |
| Rwanda | Alcohol use | -3.59 | -4.01 | -3.16 | <0.001 |
| Rwanda | All cause | -3.16 | -3.55 | -2.77 | <0.001 |
| Rwanda | Smoking | -2.81 | -3.18 | -2.44 | <0.001 |
| Saint Kitts | Alcohol use | -1.62 | -1.93 | -1.31 | <0.001 |
| Saint Kitts | All cause | -0.44 | -0.67 | -0.21 | <0.001 |
| Saint Kitts | Smoking | -0.83 | -1.08 | -0.58 | <0.001 |
| Saint Lucia | Alcohol use | -0.73 | -0.87 | -0.58 | <0.001 |
| Saint Lucia | All cause | -0.58 | -0.77 | -0.39 | <0.001 |
| Saint Lucia | Smoking | -0.86 | -1.03 | -0.69 | <0.001 |
| Saint Vincent | Alcohol use | 1.53 | 1.28 | 1.79 | <0.001 |
| Saint Vincent | All cause | 0.56 | 0.35 | 0.76 | <0.001 |
| Saint Vincent | Smoking | 0.5 | 0.29 | 0.71 | <0.001 |
| Samoa | Alcohol use | -1.06 | -1.38 | -0.73 | <0.001 |
| Samoa | All cause | -0.9 | -0.95 | -0.85 | <0.001 |
| Samoa | Smoking | -1.14 | -1.19 | -1.1 | <0.001 |
| San Marino | Alcohol use | -1.57 | -1.83 | -1.3 | <0.001 |
| San Marino | All cause | -1.59 | -1.86 | -1.33 | <0.001 |
| San Marino | Smoking | -2 | -2.28 | -1.71 | <0.001 |
| Sao Tome and Principe | Alcohol use | 1.64 | 1.35 | 1.93 | <0.001 |
| Sao Tome and Principe | All cause | 0.95 | 0.84 | 1.06 | <0.001 |
| Sao Tome and Principe | Smoking | 1.34 | 1.13 | 1.56 | <0.001 |
| Saudi Arabia | Alcohol use | -1.57 | -1.99 | -1.15 | <0.001 |
| Saudi Arabia | All cause | -1.34 | -1.51 | -1.16 | <0.001 |
| Saudi Arabia | Smoking | -1.22 | -1.4 | -1.04 | <0.001 |
| Senegal | Alcohol use | -0.38 | -0.75 | 0 | 0.048 |
| Senegal | All cause | 0.44 | 0.17 | 0.71 | 0.003 |
| Senegal | Smoking | -0.32 | -0.59 | -0.05 | 0.022 |
| Serbia | Alcohol use | -1.33 | -1.59 | -1.08 | <0.001 |
| Serbia | All cause | -1.18 | -1.45 | -0.91 | <0.001 |
| Serbia | Smoking | -1.23 | -1.53 | -0.94 | <0.001 |
| Seychelles | Alcohol use | 0.77 | 0.56 | 0.99 | <0.001 |
| Seychelles | All cause | -0.28 | -0.62 | 0.05 | 0.094 |
| Seychelles | Smoking | -0.38 | -0.69 | -0.07 | 0.018 |
| Sierra Leone | Alcohol use | -0.21 | -0.38 | -0.03 | 0.021 |
| Sierra Leone | All cause | 0.34 | 0.13 | 0.56 | 0.002 |
| Sierra Leone | Smoking | -0.03 | -0.27 | 0.21 | 0.783 |
| Singapore | Alcohol use | -4.78 | -5.17 | -4.39 | <0.001 |
| Singapore | All cause | -5.16 | -5.43 | -4.89 | <0.001 |
| Singapore | Smoking | -5.85 | -6.1 | -5.6 | <0.001 |
| Slovakia | Alcohol use | -2.92 | -3.06 | -2.77 | <0.001 |
| Slovakia | All cause | -2.62 | -2.72 | -2.52 | <0.001 |
| Slovakia | Smoking | -2.85 | -2.96 | -2.73 | <0.001 |
| Slovenia | Alcohol use | -4.18 | -4.39 | -3.97 | <0.001 |
| Slovenia | All cause | -2.83 | -2.99 | -2.67 | <0.001 |
| Slovenia | Smoking | -2.95 | -3.1 | -2.79 | <0.001 |
| Solomon Islands | Alcohol use | 1.38 | 1.08 | 1.68 | <0.001 |
| Solomon Islands | All cause | -0.4 | -0.46 | -0.35 | <0.001 |
| Solomon Islands | Smoking | -0.53 | -0.58 | -0.48 | <0.001 |
| Somalia | Alcohol use | 0 | 0 | 0 | 0.094 |
| Somalia | All cause | -0.88 | -0.91 | -0.85 | <0.001 |
| Somalia | Smoking | -1.13 | -1.17 | -1.1 | <0.001 |
| South Africa | Alcohol use | -1.89 | -2.5 | -1.28 | <0.001 |
| South Africa | All cause | -1.41 | -1.97 | -0.84 | <0.001 |
| South Africa | Smoking | -2.3 | -2.85 | -1.74 | <0.001 |
| South Korea | Alcohol use | -6.15 | -6.73 | -5.56 | <0.001 |
| South Korea | All cause | -6.45 | -7.07 | -5.83 | <0.001 |
| South Korea | Smoking | -6.51 | -7.12 | -5.89 | <0.001 |
| South Sudan | Alcohol use | -1.12 | -1.33 | -0.9 | <0.001 |
| South Sudan | All cause | -1.2 | -1.26 | -1.14 | <0.001 |
| South Sudan | Smoking | -1.48 | -1.55 | -1.41 | <0.001 |
| Spain | Alcohol use | -4.16 | -4.31 | -4 | <0.001 |
| Spain | All cause | -3.58 | -3.73 | -3.44 | <0.001 |
| Spain | Smoking | -3.97 | -4.1 | -3.84 | <0.001 |
| Sri Lanka | Alcohol use | 2.57 | 1.93 | 3.22 | <0.001 |
| Sri Lanka | All cause | 1.17 | 0.73 | 1.61 | <0.001 |
| Sri Lanka | Smoking | 0.12 | -0.29 | 0.53 | 0.544 |
| Sudan | Alcohol use | -6.36 | -8.51 | -4.16 | <0.001 |
| Sudan | All cause | -0.27 | -0.32 | -0.22 | <0.001 |
| Sudan | Smoking | -0.31 | -0.4 | -0.23 | <0.001 |
| Suriname | Alcohol use | 0.29 | 0.01 | 0.57 | 0.046 |
| Suriname | All cause | 0.12 | -0.18 | 0.43 | 0.413 |
| Suriname | Smoking | -0.14 | -0.46 | 0.18 | 0.386 |
| Swaziland | Alcohol use | 0.03 | -0.44 | 0.51 | 0.89 |
| Swaziland | All cause | 0.02 | -0.44 | 0.49 | 0.92 |
| Swaziland | Smoking | -0.82 | -1.23 | -0.41 | <0.001 |
| Sweden | Alcohol use | -1.43 | -1.55 | -1.31 | <0.001 |
| Sweden | All cause | -1.56 | -1.64 | -1.48 | <0.001 |
| Sweden | Smoking | -1.87 | -1.96 | -1.78 | <0.001 |
| Switzerland | Alcohol use | -3.49 | -3.77 | -3.21 | <0.001 |
| Switzerland | All cause | -3.12 | -3.38 | -2.85 | <0.001 |
| Switzerland | Smoking | -3.38 | -3.65 | -3.1 | <0.001 |
| Syria | Alcohol use | -1.99 | -2.24 | -1.75 | <0.001 |
| Syria | All cause | -0.91 | -1.2 | -0.61 | <0.001 |
| Syria | Smoking | -1.07 | -1.36 | -0.78 | <0.001 |
| Taiwan (CHINA) | Alcohol use | -3.51 | -3.9 | -3.11 | <0.001 |
| Taiwan (CHINA) | All cause | -2.32 | -2.59 | -2.04 | <0.001 |
| Taiwan (CHINA) | Smoking | -2.58 | -2.88 | -2.28 | <0.001 |
| Tajikistan | Alcohol use | -4.59 | -5.05 | -4.13 | <0.001 |
| Tajikistan | All cause | -1.87 | -2.11 | -1.63 | <0.001 |
| Tajikistan | Smoking | -2.77 | -3.02 | -2.51 | <0.001 |
| Tanzania | Alcohol use | -0.8 | -0.85 | -0.75 | <0.001 |
| Tanzania | All cause | -0.91 | -0.96 | -0.87 | <0.001 |
| Tanzania | Smoking | -1.33 | -1.4 | -1.26 | <0.001 |
| Thailand | Alcohol use | -1.79 | -2 | -1.58 | <0.001 |
| Thailand | All cause | -2.48 | -2.71 | -2.24 | <0.001 |
| Thailand | Smoking | -2.81 | -3.06 | -2.56 | <0.001 |
| Timor-Leste | Alcohol use | 2.49 | 2.17 | 2.81 | <0.001 |
| Timor-Leste | All cause | -0.37 | -0.68 | -0.05 | 0.024 |
| Timor-Leste | Smoking | -0.31 | -0.62 | -0.01 | 0.045 |
| Togo | Alcohol use | 0.36 | 0.05 | 0.67 | 0.025 |
| Togo | All cause | 0.54 | 0.39 | 0.7 | <0.001 |
| Togo | Smoking | -0.04 | -0.19 | 0.11 | 0.586 |
| Tokelau | Alcohol use | -0.33 | -0.38 | -0.28 | <0.001 |
| Tokelau | All cause | -1.29 | -1.35 | -1.23 | <0.001 |
| Tokelau | Smoking | -1.5 | -1.55 | -1.44 | <0.001 |
| Tonga | Alcohol use | 0.79 | 0.02 | 1.56 | 0.045 |
| Tonga | All cause | -0.56 | -0.81 | -0.32 | <0.001 |
| Tonga | Smoking | -0.79 | -1.06 | -0.52 | <0.001 |
| Trinidad | Alcohol use | -0.86 | -1.07 | -0.66 | <0.001 |
| Trinidad | All cause | -1.73 | -1.94 | -1.52 | <0.001 |
| Trinidad | Smoking | -2.05 | -2.26 | -1.84 | <0.001 |
| Tunisia | Alcohol use | 1.32 | 1.19 | 1.45 | <0.001 |
| Tunisia | All cause | -0.88 | -0.98 | -0.78 | <0.001 |
| Tunisia | Smoking | -0.98 | -1.08 | -0.87 | <0.001 |
| Turkey | Alcohol use | -2.15 | -2.4 | -1.91 | <0.001 |
| Turkey | All cause | -1.92 | -2.2 | -1.64 | <0.001 |
| Turkey | Smoking | -2.16 | -2.42 | -1.89 | <0.001 |
| Turkmenistan | Alcohol use | -2.31 | -2.71 | -1.9 | <0.001 |
| Turkmenistan | All cause | -4.1 | -4.57 | -3.63 | <0.001 |
| Turkmenistan | Smoking | -5 | -5.38 | -4.61 | <0.001 |
| Tuvalu | Alcohol use | -0.96 | -1.25 | -0.67 | <0.001 |
| Tuvalu | All cause | -0.94 | -1 | -0.88 | <0.001 |
| Tuvalu | Smoking | -1.13 | -1.21 | -1.06 | <0.001 |
| Uganda | Alcohol use | -0.54 | -0.79 | -0.29 | <0.001 |
| Uganda | All cause | -0.95 | -1.17 | -0.73 | <0.001 |
| Uganda | Smoking | -1.46 | -1.77 | -1.15 | <0.001 |
| Ukraine | Alcohol use | -2.02 | -2.39 | -1.64 | <0.001 |
| Ukraine | All cause | -2.78 | -3.27 | -2.29 | <0.001 |
| Ukraine | Smoking | -2.97 | -3.45 | -2.49 | <0.001 |
| United Arab Emirates | Alcohol use | -3.75 | -3.88 | -3.61 | <0.001 |
| United Arab Emirates | All cause | -0.77 | -1.09 | -0.45 | <0.001 |
| United Arab Emirates | Smoking | -1.03 | -1.36 | -0.71 | <0.001 |
| United Kingdom | Alcohol use | -1.61 | -1.75 | -1.47 | <0.001 |
| United Kingdom | All cause | -1.76 | -1.88 | -1.64 | <0.001 |
| United Kingdom | Smoking | -2.28 | -2.39 | -2.16 | <0.001 |
| Uruguay | Alcohol use | -2.12 | -2.31 | -1.93 | <0.001 |
| Uruguay | All cause | -2.12 | -2.28 | -1.97 | <0.001 |
| Uruguay | Smoking | -2.49 | -2.64 | -2.33 | <0.001 |
| USA | Alcohol use | -1.37 | -1.49 | -1.25 | <0.001 |
| USA | All cause | -1.79 | -1.91 | -1.67 | <0.001 |
| USA | Smoking | -2.14 | -2.26 | -2.02 | <0.001 |
| Uzbekistan | Alcohol use | -2.42 | -3.08 | -1.76 | <0.001 |
| Uzbekistan | All cause | -1.35 | -2 | -0.69 | <0.001 |
| Uzbekistan | Smoking | -2.02 | -2.64 | -1.4 | <0.001 |
| Vanuatu | Alcohol use | -0.63 | -1.23 | -0.02 | 0.042 |
| Vanuatu | All cause | -0.58 | -0.72 | -0.45 | <0.001 |
| Vanuatu | Smoking | -1.1 | -1.24 | -0.97 | <0.001 |
| Venezuela | Alcohol use | -1.33 | -1.67 | -0.99 | <0.001 |
| Venezuela | All cause | -0.89 | -1.14 | -0.64 | <0.001 |
| Venezuela | Smoking | -1.58 | -1.85 | -1.32 | <0.001 |
| Vietnam | Alcohol use | 9.64 | 8.8 | 10.49 | <0.001 |
| Vietnam | All cause | 0.93 | 0.87 | 1 | <0.001 |
| Vietnam | Smoking | 0.84 | 0.78 | 0.9 | <0.001 |
| Virgin Islands | Alcohol use | 1.94 | 1.49 | 2.4 | <0.001 |
| Virgin Islands | All cause | 1.35 | 0.97 | 1.74 | <0.001 |
| Virgin Islands | Smoking | 1.03 | 0.65 | 1.42 | <0.001 |
| Yemen | Alcohol use | -3.2 | -3.51 | -2.89 | <0.001 |
| Yemen | All cause | 0.08 | 0.03 | 0.14 | 0.004 |
| Yemen | Smoking | 0.11 | 0.06 | 0.16 | <0.001 |
| Zambia | Alcohol use | -0.23 | -0.3 | -0.17 | <0.001 |
| Zambia | All cause | -1 | -1.26 | -0.75 | <0.001 |
| Zambia | Smoking | -0.97 | -1.27 | -0.67 | <0.001 |
| Zimbabwe | Alcohol use | -0.83 | -1.08 | -0.58 | <0.001 |
| Zimbabwe | All cause | -0.03 | -0.15 | 0.09 | 0.585 |
| Zimbabwe | Smoking | -0.47 | -0.59 | -0.35 | <0.001 |

Table S2. The estimate average percentage changes (EAPCs) in age-standardized mortality rate of laryngeal cancer, 1990-2019, by location and risk factor in the sensitivity analysis.

| Location name | Risk factors | EAPCs | 95% CI | | P value |
| --- | --- | --- | --- | --- | --- |
| Afghanistan | All cause | -0.42 | -0.57 | -0.27 | <0.001 |
| Afghanistan | Smoking | 0.22 | -0.02 | 0.46 | 0.073 |
| Afghanistan | Alcohol use | 3.9 | 3.35 | 4.45 | <0.001 |
| Albania | All cause | -1.71 | -2.05 | -1.37 | <0.001 |
| Albania | Smoking | -1.53 | -1.87 | -1.19 | <0.001 |
| Albania | Alcohol use | 1.75 | 1.09 | 2.41 | <0.001 |
| Algeria | All cause | -2.01 | -2.24 | -1.77 | <0.001 |
| Algeria | Smoking | -2.17 | -2.42 | -1.92 | <0.001 |
| Algeria | Alcohol use | 0.7 | 0.41 | 1 | <0.001 |
| American Samoa | All cause | -2.43 | -2.67 | -2.19 | <0.001 |
| American Samoa | Smoking | -2.79 | -3.04 | -2.54 | <0.001 |
| American Samoa | Alcohol use | -2.59 | -3.2 | -1.99 | <0.001 |
| Andean Latin America | All cause | -1.98 | -2.24 | -1.72 | <0.001 |
| Andean Latin America | Smoking | -2.23 | -2.44 | -2.01 | <0.001 |
| Andean Latin America | Alcohol use | -1.45 | -1.83 | -1.07 | <0.001 |
| Andorra | All cause | -2.08 | -2.15 | -2.02 | <0.001 |
| Andorra | Smoking | -2.4 | -2.47 | -2.33 | <0.001 |
| Andorra | Alcohol use | -2.36 | -2.47 | -2.26 | <0.001 |
| Angola | All cause | -0.6 | -0.67 | -0.53 | <0.001 |
| Angola | Smoking | -0.69 | -0.83 | -0.56 | <0.001 |
| Angola | Alcohol use | 3.24 | 2.8 | 3.68 | <0.001 |
| Antigua | All cause | -0.1 | -0.44 | 0.24 | 0.534 |
| Antigua | Smoking | -0.16 | -0.49 | 0.16 | 0.31 |
| Antigua | Alcohol use | 2.22 | 1.76 | 2.69 | <0.001 |
| Argentina | All cause | -2.14 | -2.38 | -1.89 | <0.001 |
| Argentina | Smoking | -2.45 | -2.71 | -2.19 | <0.001 |
| Argentina | Alcohol use | -2.78 | -2.98 | -2.59 | <0.001 |
| Armenia | All cause | -2.22 | -2.41 | -2.03 | <0.001 |
| Armenia | Smoking | -2.34 | -2.52 | -2.16 | <0.001 |
| Armenia | Alcohol use | -0.7 | -1 | -0.4 | <0.001 |
| Australasia | All cause | -2.83 | -2.95 | -2.71 | <0.001 |
| Australasia | Smoking | -3.98 | -4.11 | -3.85 | <0.001 |
| Australasia | Alcohol use | -2.98 | -3.12 | -2.83 | <0.001 |
| Australia | All cause | -2.89 | -3.02 | -2.77 | <0.001 |
| Australia | Smoking | -4.1 | -4.24 | -3.96 | <0.001 |
| Australia | Alcohol use | -3.02 | -3.16 | -2.88 | <0.001 |
| Austria | All cause | -2.77 | -2.87 | -2.68 | <0.001 |
| Austria | Smoking | -2.86 | -2.96 | -2.76 | <0.001 |
| Austria | Alcohol use | -2.98 | -3.09 | -2.88 | <0.001 |
| Azerbaijan | All cause | -1.53 | -1.78 | -1.28 | <0.001 |
| Azerbaijan | Smoking | -1.5 | -1.73 | -1.27 | <0.001 |
| Azerbaijan | Alcohol use | -1.55 | -1.97 | -1.13 | <0.001 |
| Bahamas | All cause | -0.37 | -0.48 | -0.26 | <0.001 |
| Bahamas | Smoking | -0.5 | -0.63 | -0.38 | <0.001 |
| Bahamas | Alcohol use | -1.66 | -1.85 | -1.46 | <0.001 |
| Bahrain | All cause | -4.23 | -4.72 | -3.73 | <0.001 |
| Bahrain | Smoking | -4.26 | -4.74 | -3.78 | <0.001 |
| Bahrain | Alcohol use | -6.89 | -7.47 | -6.3 | <0.001 |
| Bangladesh | All cause | -2.02 | -2.22 | -1.81 | <0.001 |
| Bangladesh | Smoking | -2.31 | -2.53 | -2.1 | <0.001 |
| Bangladesh | Alcohol use | 2.36 | 1.99 | 2.72 | <0.001 |
| Barbados | All cause | -0.13 | -0.31 | 0.05 | 0.144 |
| Barbados | Smoking | -0.57 | -0.79 | -0.35 | <0.001 |
| Barbados | Alcohol use | 0.57 | 0.41 | 0.74 | <0.001 |
| Belarus | All cause | -3.08 | -3.68 | -2.48 | <0.001 |
| Belarus | Smoking | -3.1 | -3.67 | -2.53 | <0.001 |
| Belarus | Alcohol use | -2.96 | -3.5 | -2.42 | <0.001 |
| Belgium | All cause | -3.45 | -3.57 | -3.33 | <0.001 |
| Belgium | Smoking | -3.76 | -3.87 | -3.64 | <0.001 |
| Belgium | Alcohol use | -3.57 | -3.68 | -3.45 | <0.001 |
| Belize | All cause | 0.89 | 0.32 | 1.46 | 0.003 |
| Belize | Smoking | 1 | 0.46 | 1.55 | 0.001 |
| Belize | Alcohol use | 1.46 | 0.93 | 1.99 | <0.001 |
| Benin | All cause | 0.34 | 0.2 | 0.48 | <0.001 |
| Benin | Smoking | -0.44 | -0.57 | -0.32 | <0.001 |
| Benin | Alcohol use | 1.32 | 1.11 | 1.54 | <0.001 |
| Bermuda | All cause | -1.3 | -1.46 | -1.14 | <0.001 |
| Bermuda | Smoking | -1.28 | -1.43 | -1.14 | <0.001 |
| Bermuda | Alcohol use | -1.57 | -1.75 | -1.39 | <0.001 |
| Bhutan | All cause | -0.88 | -0.94 | -0.82 | <0.001 |
| Bhutan | Smoking | -1.06 | -1.16 | -0.97 | <0.001 |
| Bhutan | Alcohol use | -5.16 | -5.63 | -4.68 | <0.001 |
| Bolivia | All cause | -0.99 | -1.19 | -0.8 | <0.001 |
| Bolivia | Smoking | -1.31 | -1.64 | -0.98 | <0.001 |
| Bolivia | Alcohol use | -0.51 | -0.82 | -0.19 | 0.003 |
| Bosnia and Herzegovina | All cause | -2.05 | -2.3 | -1.8 | <0.001 |
| Bosnia and Herzegovina | Smoking | -1.91 | -2.13 | -1.68 | <0.001 |
| Bosnia and Herzegovina | Alcohol use | -1.09 | -1.42 | -0.76 | <0.001 |
| Botswana | All cause | -0.92 | -1.48 | -0.35 | 0.003 |
| Botswana | Smoking | -1.12 | -1.68 | -0.56 | <0.001 |
| Botswana | Alcohol use | -0.85 | -1.52 | -0.17 | 0.017 |
| Brazil | All cause | -0.95 | -1.03 | -0.87 | <0.001 |
| Brazil | Smoking | -2.05 | -2.2 | -1.89 | <0.001 |
| Brazil | Alcohol use | -0.56 | -0.82 | -0.3 | <0.001 |
| Brunei | All cause | -4.7 | -5.03 | -4.38 | <0.001 |
| Brunei | Smoking | -4.9 | -5.21 | -4.58 | <0.001 |
| Brunei | Alcohol use | -5.5 | -6.93 | -4.06 | <0.001 |
| Bulgaria | All cause | 1.48 | 1.06 | 1.9 | <0.001 |
| Bulgaria | Smoking | 1.35 | 0.92 | 1.77 | <0.001 |
| Bulgaria | Alcohol use | 1.55 | 1.14 | 1.96 | <0.001 |
| Burkina Faso | All cause | 0.72 | 0.57 | 0.88 | <0.001 |
| Burkina Faso | Smoking | 0.32 | 0.18 | 0.45 | <0.001 |
| Burkina Faso | Alcohol use | 1.44 | 1.34 | 1.54 | <0.001 |
| Burundi | All cause | -2.1 | -2.27 | -1.92 | <0.001 |
| Burundi | Smoking | -2.64 | -2.99 | -2.29 | <0.001 |
| Burundi | Alcohol use | -2.97 | -3.2 | -2.75 | <0.001 |
| Cambodia | All cause | -0.62 | -0.84 | -0.41 | <0.001 |
| Cambodia | Smoking | -0.64 | -0.85 | -0.43 | <0.001 |
| Cambodia | Alcohol use | 5.03 | 4.75 | 5.32 | <0.001 |
| Cameroon | All cause | 0.99 | 0.78 | 1.19 | <0.001 |
| Cameroon | Smoking | 0.71 | 0.48 | 0.95 | <0.001 |
| Cameroon | Alcohol use | 1.59 | 1.36 | 1.82 | <0.001 |
| Canada | All cause | -2.87 | -3.02 | -2.72 | <0.001 |
| Canada | Smoking | -3.46 | -3.63 | -3.28 | <0.001 |
| Canada | Alcohol use | -2.68 | -2.86 | -2.5 | <0.001 |
| Cape Verde | All cause | -1.4 | -1.98 | -0.82 | <0.001 |
| Cape Verde | Smoking | -2.64 | -3.27 | -2 | <0.001 |
| Cape Verde | Alcohol use | -1.08 | -1.58 | -0.57 | <0.001 |
| Caribbean | All cause | 0.04 | -0.05 | 0.13 | 0.353 |
| Caribbean | Smoking | -0.12 | -0.2 | -0.04 | 0.007 |
| Caribbean | Alcohol use | 0.6 | 0.49 | 0.72 | <0.001 |
| Central African Republic | All cause | -1.08 | -1.11 | -1.05 | <0.001 |
| Central African Republic | Smoking | -1.71 | -1.81 | -1.62 | <0.001 |
| Central African Republic | Alcohol use | -1.99 | -2.21 | -1.77 | <0.001 |
| Central Asia | All cause | -2.09 | -2.28 | -1.9 | <0.001 |
| Central Asia | Smoking | -2.35 | -2.49 | -2.21 | <0.001 |
| Central Asia | Alcohol use | -2.39 | -2.53 | -2.26 | <0.001 |
| Central Europe | All cause | -1.36 | -1.48 | -1.25 | <0.001 |
| Central Europe | Smoking | -1.54 | -1.65 | -1.42 | <0.001 |
| Central Europe | Alcohol use | -1.36 | -1.45 | -1.26 | <0.001 |
| Central Latin America | All cause | -2.4 | -2.53 | -2.28 | <0.001 |
| Central Latin America | Smoking | -3.24 | -3.39 | -3.09 | <0.001 |
| Central Latin America | Alcohol use | -2.35 | -2.5 | -2.2 | <0.001 |
| Central Sub-Saharan Africa | All cause | -0.97 | -1.06 | -0.88 | <0.001 |
| Central Sub-Saharan Africa | Smoking | -0.94 | -1.15 | -0.74 | <0.001 |
| Central Sub-Saharan Africa | Alcohol use | 0.08 | -0.42 | 0.59 | 0.745 |
| Chad | All cause | 1.43 | 1.22 | 1.64 | <0.001 |
| Chad | Smoking | 0.87 | 0.6 | 1.14 | <0.001 |
| Chad | Alcohol use | 4.15 | 3.83 | 4.46 | <0.001 |
| Chile | All cause | -2.32 | -2.47 | -2.17 | <0.001 |
| Chile | Smoking | -3.07 | -3.17 | -2.98 | <0.001 |
| Chile | Alcohol use | -2.21 | -2.43 | -1.99 | <0.001 |
| China | All cause | -0.95 | -1.06 | -0.83 | <0.001 |
| China | Smoking | -0.83 | -0.95 | -0.72 | <0.001 |
| China | Alcohol use | -0.32 | -0.55 | -0.1 | 0.006 |
| Colombia | All cause | -3.67 | -3.93 | -3.42 | <0.001 |
| Colombia | Smoking | -4.8 | -5.06 | -4.54 | <0.001 |
| Colombia | Alcohol use | -4.08 | -4.42 | -3.73 | <0.001 |
| Comoros | All cause | -1.01 | -1.21 | -0.82 | <0.001 |
| Comoros | Smoking | -1.35 | -1.54 | -1.16 | <0.001 |
| Comoros | Alcohol use | 1.22 | 1.07 | 1.38 | <0.001 |
| Cook Islands | All cause | -1.74 | -2.01 | -1.47 | <0.001 |
| Cook Islands | Smoking | -2.1 | -2.37 | -1.83 | <0.001 |
| Cook Islands | Alcohol use | 4.13 | 3.18 | 5.1 | <0.001 |
| Costa Rica | All cause | -1.82 | -2 | -1.63 | <0.001 |
| Costa Rica | Smoking | -2.39 | -2.57 | -2.22 | <0.001 |
| Costa Rica | Alcohol use | -2.79 | -3.12 | -2.46 | <0.001 |
| Croatia | All cause | -2.57 | -2.72 | -2.41 | <0.001 |
| Croatia | Smoking | -2.66 | -2.8 | -2.51 | <0.001 |
| Croatia | Alcohol use | -2.78 | -2.94 | -2.61 | <0.001 |
| Cuba | All cause | 0.72 | 0.62 | 0.81 | <0.001 |
| Cuba | Smoking | 0.5 | 0.38 | 0.61 | <0.001 |
| Cuba | Alcohol use | 1.71 | 1.57 | 1.86 | <0.001 |
| Cyprus | All cause | -1.86 | -2.06 | -1.65 | <0.001 |
| Cyprus | Smoking | -1.88 | -2.1 | -1.65 | <0.001 |
| Cyprus | Alcohol use | -1.66 | -1.94 | -1.37 | <0.001 |
| Czech Republic | All cause | -2.31 | -2.39 | -2.23 | <0.001 |
| Czech Republic | Smoking | -2.53 | -2.6 | -2.45 | <0.001 |
| Czech Republic | Alcohol use | -2.45 | -2.54 | -2.37 | <0.001 |
| C么te d'Ivoire | All cause | 0.16 | 0.01 | 0.32 | 0.041 |
| C么te d'Ivoire | Smoking | 0.29 | 0.05 | 0.52 | 0.019 |
| C么te d'Ivoire | Alcohol use | 0.85 | 0.68 | 1.01 | <0.001 |
| Democratic Republic of the Congo | All cause | -1.02 | -1.12 | -0.93 | <0.001 |
| Democratic Republic of the Congo | Smoking | -1.11 | -1.33 | -0.89 | <0.001 |
| Democratic Republic of the Congo | Alcohol use | -1.59 | -2.6 | -0.57 | 0.004 |
| Denmark | All cause | -2.95 | -3.04 | -2.86 | <0.001 |
| Denmark | Smoking | -3.38 | -3.48 | -3.28 | <0.001 |
| Denmark | Alcohol use | -3.45 | -3.58 | -3.32 | <0.001 |
| Djibouti | All cause | -0.62 | -0.74 | -0.5 | <0.001 |
| Djibouti | Smoking | -0.3 | -0.42 | -0.19 | <0.001 |
| Djibouti | Alcohol use | -3.75 | -3.9 | -3.6 | <0.001 |
| Dominica | All cause | 0.43 | 0.35 | 0.51 | <0.001 |
| Dominica | Smoking | 0.3 | 0.17 | 0.42 | <0.001 |
| Dominica | Alcohol use | 0.6 | 0.49 | 0.7 | <0.001 |
| Dominican Republic | All cause | 1.18 | 0.89 | 1.48 | <0.001 |
| Dominican Republic | Smoking | 0.99 | 0.75 | 1.23 | <0.001 |
| Dominican Republic | Alcohol use | 1.37 | 1.17 | 1.57 | <0.001 |
| East Asia | All cause | -0.96 | -1.07 | -0.86 | <0.001 |
| East Asia | Smoking | -0.85 | -0.96 | -0.75 | <0.001 |
| East Asia | Alcohol use | -0.39 | -0.61 | -0.17 | 0.001 |
| Eastern Europe | All cause | -2.79 | -3.17 | -2.41 | <0.001 |
| Eastern Europe | Smoking | -2.85 | -3.23 | -2.46 | <0.001 |
| Eastern Europe | Alcohol use | -2.58 | -2.97 | -2.19 | <0.001 |
| Eastern Sub-Saharan Africa | All cause | -1.13 | -1.18 | -1.09 | <0.001 |
| Eastern Sub-Saharan Africa | Smoking | -1.28 | -1.33 | -1.24 | <0.001 |
| Eastern Sub-Saharan Africa | Alcohol use | -0.77 | -0.86 | -0.68 | <0.001 |
| Ecuador | All cause | -0.64 | -0.91 | -0.38 | <0.001 |
| Ecuador | Smoking | -1.47 | -1.74 | -1.19 | <0.001 |
| Ecuador | Alcohol use | 0.55 | 0.2 | 0.91 | 0.004 |
| Egypt | All cause | 0.58 | 0.37 | 0.8 | <0.001 |
| Egypt | Smoking | 0.81 | 0.53 | 1.08 | <0.001 |
| Egypt | Alcohol use | 0.94 | 0.73 | 1.16 | <0.001 |
| El Salvador | All cause | -0.17 | -0.45 | 0.11 | 0.223 |
| El Salvador | Smoking | 0.06 | -0.22 | 0.34 | 0.686 |
| El Salvador | Alcohol use | -0.05 | -0.39 | 0.3 | 0.781 |
| Equatorial Guinea | All cause | -1.85 | -2.28 | -1.42 | <0.001 |
| Equatorial Guinea | Smoking | -1.82 | -2.37 | -1.28 | <0.001 |
| Equatorial Guinea | Alcohol use | 0.88 | 0.65 | 1.12 | <0.001 |
| Eritrea | All cause | -0.83 | -0.98 | -0.68 | <0.001 |
| Eritrea | Smoking | -1.48 | -1.65 | -1.31 | <0.001 |
| Eritrea | Alcohol use | -1.3 | -1.74 | -0.87 | <0.001 |
| Estonia | All cause | -2.81 | -3.12 | -2.51 | <0.001 |
| Estonia | Smoking | -2.9 | -3.21 | -2.58 | <0.001 |
| Estonia | Alcohol use | -2.02 | -2.34 | -1.7 | <0.001 |
| Ethiopia | All cause | -2.63 | -2.78 | -2.49 | <0.001 |
| Ethiopia | Smoking | -2.62 | -3 | -2.24 | <0.001 |
| Ethiopia | Alcohol use | -1.24 | -1.66 | -0.81 | <0.001 |
| Fiji | All cause | -0.17 | -0.35 | 0.02 | 0.071 |
| Fiji | Smoking | -0.8 | -1.02 | -0.58 | <0.001 |
| Fiji | Alcohol use | 0.89 | 0.52 | 1.26 | <0.001 |
| Finland | All cause | -2.09 | -2.28 | -1.91 | <0.001 |
| Finland | Smoking | -2.48 | -2.66 | -2.31 | <0.001 |
| Finland | Alcohol use | -1.94 | -2.1 | -1.78 | <0.001 |
| France | All cause | -3.84 | -4.21 | -3.46 | <0.001 |
| France | Smoking | -4.09 | -4.48 | -3.7 | <0.001 |
| France | Alcohol use | -3.89 | -4.29 | -3.49 | <0.001 |
| Gabon | All cause | -1.11 | -1.16 | -1.06 | <0.001 |
| Gabon | Smoking | -0.89 | -0.94 | -0.83 | <0.001 |
| Gabon | Alcohol use | -1.39 | -1.55 | -1.24 | <0.001 |
| Gambia | All cause | 0.01 | -0.17 | 0.19 | 0.895 |
| Gambia | Smoking | -0.81 | -0.99 | -0.63 | <0.001 |
| Gambia | Alcohol use | 1.66 | 1.27 | 2.04 | <0.001 |
| Georgia | All cause | -0.47 | -0.87 | -0.07 | 0.023 |
| Georgia | Smoking | -0.17 | -0.6 | 0.25 | 0.409 |
| Georgia | Alcohol use | 1.24 | 0.61 | 1.87 | <0.001 |
| Germany | All cause | -1.9 | -1.95 | -1.84 | <0.001 |
| Germany | Smoking | -2.16 | -2.21 | -2.11 | <0.001 |
| Germany | Alcohol use | -2.09 | -2.16 | -2.02 | <0.001 |
| Ghana | All cause | 1.33 | 1.08 | 1.57 | <0.001 |
| Ghana | Smoking | 1.48 | 1.17 | 1.78 | <0.001 |
| Ghana | Alcohol use | 1.86 | 1.61 | 2.1 | <0.001 |
| Global | All cause | -1.51 | -1.59 | -1.43 | <0.001 |
| Global | Smoking | -1.93 | -2.01 | -1.85 | <0.001 |
| Global | Alcohol use | -1.77 | -1.89 | -1.65 | <0.001 |
| Greece | All cause | -1.2 | -1.28 | -1.13 | <0.001 |
| Greece | Smoking | -1.27 | -1.34 | -1.19 | <0.001 |
| Greece | Alcohol use | -1.54 | -1.64 | -1.44 | <0.001 |
| Greenland | All cause | -1.29 | -1.5 | -1.09 | <0.001 |
| Greenland | Smoking | -1.57 | -1.76 | -1.38 | <0.001 |
| Greenland | Alcohol use | -1.03 | -1.31 | -0.75 | <0.001 |
| Grenada | All cause | -0.75 | -1.15 | -0.34 | 0.001 |
| Grenada | Smoking | -1.04 | -1.48 | -0.6 | <0.001 |
| Grenada | Alcohol use | -0.54 | -0.96 | -0.11 | 0.016 |
| Guam | All cause | -3.13 | -3.49 | -2.76 | <0.001 |
| Guam | Smoking | -3.38 | -3.73 | -3.02 | <0.001 |
| Guam | Alcohol use | -0.97 | -1.42 | -0.52 | <0.001 |
| Guatemala | All cause | -2.33 | -2.6 | -2.07 | <0.001 |
| Guatemala | Smoking | -2.79 | -3.01 | -2.57 | <0.001 |
| Guatemala | Alcohol use | -2.47 | -2.76 | -2.18 | <0.001 |
| Guinea | All cause | 1.74 | 1.53 | 1.94 | <0.001 |
| Guinea | Smoking | 1.99 | 1.76 | 2.23 | <0.001 |
| Guinea | Alcohol use | 2.53 | 2.43 | 2.63 | <0.001 |
| Guinea-Bissau | All cause | 0.07 | -0.12 | 0.25 | 0.471 |
| Guinea-Bissau | Smoking | 0.92 | 0.57 | 1.26 | <0.001 |
| Guinea-Bissau | Alcohol use | 0.06 | -0.16 | 0.28 | 0.572 |
| Guyana | All cause | -0.1 | -0.2 | 0 | 0.042 |
| Guyana | Smoking | -0.38 | -0.47 | -0.28 | <0.001 |
| Guyana | Alcohol use | -0.52 | -0.61 | -0.43 | <0.001 |
| Haiti | All cause | -0.55 | -0.7 | -0.41 | <0.001 |
| Haiti | Smoking | -1.61 | -1.81 | -1.41 | <0.001 |
| Haiti | Alcohol use | -0.52 | -0.66 | -0.38 | <0.001 |
| High SDI | All cause | -2.6 | -2.74 | -2.46 | <0.001 |
| High SDI | Smoking | -2.94 | -3.08 | -2.8 | <0.001 |
| High SDI | Alcohol use | -2.76 | -2.92 | -2.59 | <0.001 |
| High-income Asia Pacific | All cause | -3.68 | -3.94 | -3.42 | <0.001 |
| High-income Asia Pacific | Smoking | -4.03 | -4.31 | -3.76 | <0.001 |
| High-income Asia Pacific | Alcohol use | -3.83 | -4.15 | -3.51 | <0.001 |
| High-income North America | All cause | -1.9 | -2.02 | -1.78 | <0.001 |
| High-income North America | Smoking | -2.26 | -2.39 | -2.14 | <0.001 |
| High-income North America | Alcohol use | -1.49 | -1.62 | -1.36 | <0.001 |
| High-middle SDI | All cause | -2.49 | -2.63 | -2.34 | <0.001 |
| High-middle SDI | Smoking | -2.76 | -2.91 | -2.61 | <0.001 |
| High-middle SDI | Alcohol use | -2.8 | -2.96 | -2.64 | <0.001 |
| Honduras | All cause | 0.91 | 0.71 | 1.11 | <0.001 |
| Honduras | Smoking | 0.74 | 0.52 | 0.96 | <0.001 |
| Honduras | Alcohol use | 0.95 | 0.82 | 1.08 | <0.001 |
| Hungary | All cause | -1.68 | -1.94 | -1.42 | <0.001 |
| Hungary | Smoking | -1.88 | -2.16 | -1.59 | <0.001 |
| Hungary | Alcohol use | -2.15 | -2.39 | -1.91 | <0.001 |
| Iceland | All cause | -2.33 | -2.55 | -2.11 | <0.001 |
| Iceland | Smoking | -2.81 | -3.03 | -2.59 | <0.001 |
| Iceland | Alcohol use | -0.84 | -0.99 | -0.7 | <0.001 |
| India | All cause | -1.11 | -1.24 | -0.98 | <0.001 |
| India | Smoking | -1.62 | -1.74 | -1.5 | <0.001 |
| India | Alcohol use | 0.55 | 0.22 | 0.87 | 0.002 |
| Indonesia | All cause | -0.04 | -0.12 | 0.04 | 0.325 |
| Indonesia | Smoking | 0.23 | 0.13 | 0.33 | <0.001 |
| Indonesia | Alcohol use | -0.35 | -0.52 | -0.18 | <0.001 |
| Iran | All cause | -1.28 | -1.5 | -1.07 | <0.001 |
| Iran | Smoking | -1.24 | -1.37 | -1.1 | <0.001 |
| Iran | Alcohol use | 3.73 | 3.17 | 4.29 | <0.001 |
| Iraq | All cause | -0.53 | -0.63 | -0.43 | <0.001 |
| Iraq | Smoking | -0.73 | -0.83 | -0.62 | <0.001 |
| Iraq | Alcohol use | -3.22 | -3.73 | -2.7 | <0.001 |
| Ireland | All cause | -2.2 | -2.34 | -2.06 | <0.001 |
| Ireland | Smoking | -2.77 | -2.94 | -2.6 | <0.001 |
| Ireland | Alcohol use | -2.32 | -2.55 | -2.09 | <0.001 |
| Israel | All cause | -1.32 | -1.6 | -1.04 | <0.001 |
| Israel | Smoking | -1.66 | -1.95 | -1.36 | <0.001 |
| Israel | Alcohol use | 0.74 | 0.57 | 0.9 | <0.001 |
| Italy | All cause | -3.2 | -3.32 | -3.08 | <0.001 |
| Italy | Smoking | -3.59 | -3.72 | -3.46 | <0.001 |
| Italy | Alcohol use | -3.79 | -3.95 | -3.64 | <0.001 |
| Jamaica | All cause | 0.91 | 0.42 | 1.4 | 0.001 |
| Jamaica | Smoking | 0.5 | -0.04 | 1.05 | 0.068 |
| Jamaica | Alcohol use | 1.61 | 1.13 | 2.09 | <0.001 |
| Japan | All cause | -2.51 | -2.59 | -2.43 | <0.001 |
| Japan | Smoking | -2.97 | -3.07 | -2.88 | <0.001 |
| Japan | Alcohol use | -2.78 | -2.92 | -2.64 | <0.001 |
| Jordan | All cause | -4.38 | -5.04 | -3.72 | <0.001 |
| Jordan | Smoking | -4.36 | -5 | -3.73 | <0.001 |
| Jordan | Alcohol use | -2.59 | -3.48 | -1.7 | <0.001 |
| Kazakhstan | All cause | -3.35 | -3.59 | -3.12 | <0.001 |
| Kazakhstan | Smoking | -3.37 | -3.62 | -3.13 | <0.001 |
| Kazakhstan | Alcohol use | -4.18 | -4.48 | -3.88 | <0.001 |
| Kenya | All cause | 0.82 | 0.51 | 1.12 | <0.001 |
| Kenya | Smoking | -0.05 | -0.36 | 0.26 | 0.748 |
| Kenya | Alcohol use | 0.34 | 0.13 | 0.56 | 0.003 |
| Kiribati | All cause | -0.38 | -0.44 | -0.33 | <0.001 |
| Kiribati | Smoking | -0.35 | -0.4 | -0.29 | <0.001 |
| Kiribati | Alcohol use | -1.16 | -1.33 | -0.98 | <0.001 |
| Kuwait | All cause | -1.46 | -1.86 | -1.06 | <0.001 |
| Kuwait | Smoking | -1.44 | -1.82 | -1.06 | <0.001 |
| Kuwait | Alcohol use | 0.68 | 0.56 | 0.8 | <0.001 |
| Kyrgyzstan | All cause | -3.72 | -4.14 | -3.3 | <0.001 |
| Kyrgyzstan | Smoking | -3.68 | -4.11 | -3.25 | <0.001 |
| Kyrgyzstan | Alcohol use | -3.02 | -3.34 | -2.69 | <0.001 |
| Laos | All cause | -2.16 | -2.33 | -1.98 | <0.001 |
| Laos | Smoking | -2.06 | -2.25 | -1.86 | <0.001 |
| Laos | Alcohol use | -0.79 | -1.28 | -0.3 | 0.002 |
| Latvia | All cause | -1.77 | -2.17 | -1.37 | <0.001 |
| Latvia | Smoking | -1.87 | -2.26 | -1.48 | <0.001 |
| Latvia | Alcohol use | -1.11 | -1.51 | -0.72 | <0.001 |
| Lebanon | All cause | -0.94 | -1.15 | -0.73 | <0.001 |
| Lebanon | Smoking | -0.76 | -0.96 | -0.56 | <0.001 |
| Lebanon | Alcohol use | -2.38 | -2.64 | -2.13 | <0.001 |
| Lesotho | All cause | 1.48 | 1.18 | 1.77 | <0.001 |
| Lesotho | Smoking | 1.4 | 1.13 | 1.66 | <0.001 |
| Lesotho | Alcohol use | 1.85 | 1.55 | 2.15 | <0.001 |
| Liberia | All cause | -0.05 | -0.26 | 0.15 | 0.589 |
| Liberia | Smoking | -0.29 | -0.5 | -0.08 | 0.008 |
| Liberia | Alcohol use | -0.44 | -0.7 | -0.18 | 0.002 |
| Libya | All cause | -0.57 | -0.77 | -0.37 | <0.001 |
| Libya | Smoking | -0.61 | -0.81 | -0.41 | <0.001 |
| Libya | Alcohol use | 2.4 | 1.38 | 3.42 | <0.001 |
| Lithuania | All cause | -1.48 | -1.85 | -1.11 | <0.001 |
| Lithuania | Smoking | -1.6 | -1.97 | -1.23 | <0.001 |
| Lithuania | Alcohol use | -0.78 | -1.2 | -0.36 | 0.001 |
| Low SDI | All cause | -0.72 | -0.78 | -0.65 | <0.001 |
| Low SDI | Smoking | -1.08 | -1.17 | -0.98 | <0.001 |
| Low SDI | Alcohol use | 0.15 | 0 | 0.3 | 0.044 |
| Low-middle SDI | All cause | -0.8 | -0.88 | -0.72 | <0.001 |
| Low-middle SDI | Smoking | -1.22 | -1.29 | -1.14 | <0.001 |
| Low-middle SDI | Alcohol use | 0.76 | 0.55 | 0.97 | <0.001 |
| Luxembourg | All cause | -3.46 | -3.54 | -3.38 | <0.001 |
| Luxembourg | Smoking | -3.83 | -3.91 | -3.75 | <0.001 |
| Luxembourg | Alcohol use | -3.71 | -3.81 | -3.61 | <0.001 |
| Macedonia | All cause | -0.23 | -0.51 | 0.06 | 0.121 |
| Macedonia | Smoking | -0.28 | -0.58 | 0.01 | 0.06 |
| Macedonia | Alcohol use | -1.01 | -1.19 | -0.83 | <0.001 |
| Madagascar | All cause | -1.04 | -1.16 | -0.92 | <0.001 |
| Madagascar | Smoking | -2.23 | -2.4 | -2.05 | <0.001 |
| Madagascar | Alcohol use | -1.55 | -1.86 | -1.24 | <0.001 |
| Malawi | All cause | -0.65 | -0.85 | -0.46 | <0.001 |
| Malawi | Smoking | -0.63 | -0.86 | -0.41 | <0.001 |
| Malawi | Alcohol use | 0.85 | 0.71 | 0.99 | <0.001 |
| Malaysia | All cause | -1.71 | -2.03 | -1.39 | <0.001 |
| Malaysia | Smoking | -1.99 | -2.31 | -1.67 | <0.001 |
| Malaysia | Alcohol use | -3.03 | -3.63 | -2.42 | <0.001 |
| Maldives | All cause | -3.23 | -3.5 | -2.96 | <0.001 |
| Maldives | Smoking | -3.19 | -3.46 | -2.91 | <0.001 |
| Maldives | Alcohol use | -1.33 | -2.8 | 0.16 | 0.077 |
| Mali | All cause | -0.31 | -0.43 | -0.2 | <0.001 |
| Mali | Smoking | 0.59 | 0.45 | 0.73 | <0.001 |
| Mali | Alcohol use | 0.23 | 0.12 | 0.35 | <0.001 |
| Malta | All cause | -2.63 | -2.74 | -2.53 | <0.001 |
| Malta | Smoking | -2.94 | -3.08 | -2.8 | <0.001 |
| Malta | Alcohol use | -2.22 | -2.42 | -2.03 | <0.001 |
| Marshall Islands | All cause | -0.28 | -0.42 | -0.13 | <0.001 |
| Marshall Islands | Smoking | -0.3 | -0.47 | -0.12 | 0.001 |
| Marshall Islands | Alcohol use | -0.01 | -0.13 | 0.11 | 0.837 |
| Mauritania | All cause | -0.37 | -0.62 | -0.12 | 0.006 |
| Mauritania | Smoking | -0.82 | -1.08 | -0.55 | <0.001 |
| Mauritania | Alcohol use | -0.27 | -0.32 | -0.23 | <0.001 |
| Mauritius | All cause | -2.46 | -2.64 | -2.28 | <0.001 |
| Mauritius | Smoking | -2.24 | -2.47 | -2.01 | <0.001 |
| Mauritius | Alcohol use | -2.35 | -2.62 | -2.08 | <0.001 |
| Mexico | All cause | -2.8 | -2.95 | -2.64 | <0.001 |
| Mexico | Smoking | -3.77 | -3.96 | -3.58 | <0.001 |
| Mexico | Alcohol use | -2.41 | -2.6 | -2.21 | <0.001 |
| Micronesia | All cause | -0.98 | -1.1 | -0.87 | <0.001 |
| Micronesia | Smoking | -1.11 | -1.23 | -0.98 | <0.001 |
| Micronesia | Alcohol use | -2.5 | -2.8 | -2.2 | <0.001 |
| Middle SDI | All cause | -0.87 | -0.92 | -0.82 | <0.001 |
| Middle SDI | Smoking | -1.1 | -1.15 | -1.05 | <0.001 |
| Middle SDI | Alcohol use | 0.02 | -0.12 | 0.16 | 0.813 |
| Monaco | All cause | -1.63 | -1.81 | -1.46 | <0.001 |
| Monaco | Smoking | -1.97 | -2.16 | -1.79 | <0.001 |
| Monaco | Alcohol use | -1.7 | -1.96 | -1.44 | <0.001 |
| Mongolia | All cause | 1.63 | 1.28 | 1.99 | <0.001 |
| Mongolia | Smoking | 2.32 | 1.94 | 2.7 | <0.001 |
| Mongolia | Alcohol use | 5 | 4.49 | 5.52 | <0.001 |
| Montenegro | All cause | -0.26 | -0.5 | -0.03 | 0.029 |
| Montenegro | Smoking | -0.34 | -0.6 | -0.07 | 0.014 |
| Montenegro | Alcohol use | -0.79 | -0.94 | -0.65 | <0.001 |
| Morocco | All cause | -0.42 | -0.65 | -0.19 | 0.001 |
| Morocco | Smoking | -0.88 | -1.14 | -0.62 | <0.001 |
| Morocco | Alcohol use | -2.01 | -2.14 | -1.87 | <0.001 |
| Mozambique | All cause | 0.92 | 0.77 | 1.07 | <0.001 |
| Mozambique | Smoking | 1 | 0.82 | 1.18 | <0.001 |
| Mozambique | Alcohol use | 4.23 | 4.07 | 4.39 | <0.001 |
| Myanmar | All cause | -2.1 | -2.19 | -2.02 | <0.001 |
| Myanmar | Smoking | -2.72 | -2.82 | -2.62 | <0.001 |
| Myanmar | Alcohol use | 4.54 | 4.22 | 4.85 | <0.001 |
| Namibia | All cause | 0.13 | -0.15 | 0.41 | 0.342 |
| Namibia | Smoking | -0.47 | -0.74 | -0.2 | 0.001 |
| Namibia | Alcohol use | 1.71 | 1.45 | 1.96 | <0.001 |
| Nauru | All cause | -0.65 | -0.96 | -0.34 | <0.001 |
| Nauru | Smoking | -0.95 | -1.34 | -0.57 | <0.001 |
| Nauru | Alcohol use | -0.3 | -0.65 | 0.06 | 0.095 |
| Nepal | All cause | -0.54 | -0.87 | -0.21 | 0.002 |
| Nepal | Smoking | -1.27 | -1.53 | -1 | <0.001 |
| Nepal | Alcohol use | 6.9 | 6.28 | 7.53 | <0.001 |
| Netherlands | All cause | -2.33 | -2.57 | -2.09 | <0.001 |
| Netherlands | Smoking | -2.87 | -3.12 | -2.62 | <0.001 |
| Netherlands | Alcohol use | -2.71 | -2.96 | -2.47 | <0.001 |
| New Zealand | All cause | -2.41 | -2.53 | -2.29 | <0.001 |
| New Zealand | Smoking | -3.2 | -3.32 | -3.07 | <0.001 |
| New Zealand | Alcohol use | -2.69 | -2.87 | -2.5 | <0.001 |
| Nicaragua | All cause | -1 | -1.22 | -0.77 | <0.001 |
| Nicaragua | Smoking | -1.21 | -1.39 | -1.03 | <0.001 |
| Nicaragua | Alcohol use | -0.05 | -0.17 | 0.06 | 0.354 |
| Niger | All cause | 0.41 | 0.23 | 0.59 | <0.001 |
| Niger | Smoking | 0.92 | 0.75 | 1.09 | <0.001 |
| Niger | Alcohol use | 0.93 | 0.47 | 1.4 | <0.001 |
| Nigeria | All cause | -0.28 | -0.48 | -0.08 | 0.008 |
| Nigeria | Smoking | -1.07 | -1.24 | -0.89 | <0.001 |
| Nigeria | Alcohol use | 0.1 | -0.19 | 0.4 | 0.482 |
| Niue | All cause | -1.03 | -1.16 | -0.9 | <0.001 |
| Niue | Smoking | -1.23 | -1.38 | -1.08 | <0.001 |
| Niue | Alcohol use | -1.52 | -1.82 | -1.23 | <0.001 |
| North Africa and Middle East | All cause | -0.94 | -1.05 | -0.82 | <0.001 |
| North Africa and Middle East | Smoking | -1.09 | -1.19 | -1 | <0.001 |
| North Africa and Middle East | Alcohol use | -1.25 | -1.35 | -1.15 | <0.001 |
| North Korea | All cause | -0.43 | -0.5 | -0.37 | <0.001 |
| North Korea | Smoking | -0.36 | -0.43 | -0.28 | <0.001 |
| North Korea | Alcohol use | -0.59 | -0.67 | -0.52 | <0.001 |
| Northern Mariana Islands | All cause | -3.13 | -3.7 | -2.56 | <0.001 |
| Northern Mariana Islands | Smoking | -3.95 | -4.51 | -3.39 | <0.001 |
| Northern Mariana Islands | Alcohol use | -3.32 | -3.92 | -2.72 | <0.001 |
| Norway | All cause | -2.15 | -2.25 | -2.05 | <0.001 |
| Norway | Smoking | -3.66 | -3.86 | -3.45 | <0.001 |
| Norway | Alcohol use | -1.52 | -1.72 | -1.32 | <0.001 |
| Oceania | All cause | -0.44 | -0.46 | -0.42 | <0.001 |
| Oceania | Smoking | -0.93 | -0.97 | -0.89 | <0.001 |
| Oceania | Alcohol use | -0.58 | -0.88 | -0.28 | 0.001 |
| Oman | All cause | -1.11 | -1.37 | -0.85 | <0.001 |
| Oman | Smoking | -1.33 | -1.54 | -1.12 | <0.001 |
| Oman | Alcohol use | -0.56 | -1.58 | 0.47 | 0.275 |
| Pakistan | All cause | -0.34 | -0.62 | -0.05 | 0.023 |
| Pakistan | Smoking | -0.99 | -1.33 | -0.65 | <0.001 |
| Pakistan | Alcohol use | 1.04 | 0.82 | 1.26 | <0.001 |
| Palau | All cause | -0.7 | -0.79 | -0.62 | <0.001 |
| Palau | Smoking | -1.17 | -1.21 | -1.13 | <0.001 |
| Palau | Alcohol use | -0.01 | -0.13 | 0.11 | 0.847 |
| Palestine | All cause | -1.74 | -1.91 | -1.58 | <0.001 |
| Palestine | Smoking | -1.79 | -1.95 | -1.62 | <0.001 |
| Palestine | Alcohol use | -0.64 | -1.02 | -0.26 | 0.002 |
| Panama | All cause | -2.6 | -2.8 | -2.39 | <0.001 |
| Panama | Smoking | -3.41 | -3.61 | -3.21 | <0.001 |
| Panama | Alcohol use | -2.15 | -2.43 | -1.87 | <0.001 |
| Papua New Guinea | All cause | -0.26 | -0.29 | -0.23 | <0.001 |
| Papua New Guinea | Smoking | -0.76 | -0.82 | -0.7 | <0.001 |
| Papua New Guinea | Alcohol use | -0.97 | -1.44 | -0.49 | <0.001 |
| Paraguay | All cause | 0.73 | 0.6 | 0.86 | <0.001 |
| Paraguay | Smoking | 0.24 | 0.09 | 0.4 | 0.003 |
| Paraguay | Alcohol use | 0.19 | 0 | 0.38 | 0.046 |
| Peru | All cause | -3.04 | -3.41 | -2.67 | <0.001 |
| Peru | Smoking | -3.65 | -3.96 | -3.33 | <0.001 |
| Peru | Alcohol use | -2.52 | -3.09 | -1.94 | <0.001 |
| Philippines | All cause | -1.14 | -1.34 | -0.93 | <0.001 |
| Philippines | Smoking | -1.44 | -1.63 | -1.24 | <0.001 |
| Philippines | Alcohol use | -0.63 | -0.84 | -0.42 | <0.001 |
| Poland | All cause | -1.97 | -2.11 | -1.82 | <0.001 |
| Poland | Smoking | -2.23 | -2.36 | -2.1 | <0.001 |
| Poland | Alcohol use | -1.8 | -1.91 | -1.69 | <0.001 |
| Portugal | All cause | -2.23 | -2.44 | -2.02 | <0.001 |
| Portugal | Smoking | -2.53 | -2.77 | -2.29 | <0.001 |
| Portugal | Alcohol use | -2.48 | -2.71 | -2.24 | <0.001 |
| Puerto Rico | All cause | -4.33 | -4.62 | -4.03 | <0.001 |
| Puerto Rico | Smoking | -4.54 | -4.86 | -4.22 | <0.001 |
| Puerto Rico | Alcohol use | -4.33 | -4.76 | -3.9 | <0.001 |
| Qatar | All cause | -1.61 | -2.03 | -1.18 | <0.001 |
| Qatar | Smoking | -1.89 | -2.33 | -1.45 | <0.001 |
| Qatar | Alcohol use | -2.92 | -3.32 | -2.52 | <0.001 |
| Republic of Congo | All cause | -1.72 | -1.95 | -1.5 | <0.001 |
| Republic of Congo | Smoking | -1.5 | -1.78 | -1.22 | <0.001 |
| Republic of Congo | Alcohol use | -0.57 | -1.3 | 0.17 | 0.125 |
| Republic of Moldova | All cause | -0.59 | -0.95 | -0.22 | 0.003 |
| Republic of Moldova | Smoking | -0.53 | -0.9 | -0.16 | 0.007 |
| Republic of Moldova | Alcohol use | -1.2 | -1.57 | -0.83 | <0.001 |
| Romania | All cause | -0.21 | -0.45 | 0.02 | 0.069 |
| Romania | Smoking | -0.35 | -0.58 | -0.12 | 0.005 |
| Romania | Alcohol use | -0.04 | -0.25 | 0.18 | 0.722 |
| Russia | All cause | -2.78 | -3.19 | -2.36 | <0.001 |
| Russia | Smoking | -2.81 | -3.23 | -2.38 | <0.001 |
| Russia | Alcohol use | -2.85 | -3.33 | -2.37 | <0.001 |
| Rwanda | All cause | -3.17 | -3.57 | -2.77 | <0.001 |
| Rwanda | Smoking | -2.8 | -3.17 | -2.43 | <0.001 |
| Rwanda | Alcohol use | -3.56 | -3.98 | -3.14 | <0.001 |
| Saint Kitts | All cause | -0.46 | -0.7 | -0.23 | <0.001 |
| Saint Kitts | Smoking | -0.84 | -1.09 | -0.58 | <0.001 |
| Saint Kitts | Alcohol use | -1.65 | -1.96 | -1.35 | <0.001 |
| Saint Lucia | All cause | -0.67 | -0.85 | -0.48 | <0.001 |
| Saint Lucia | Smoking | -0.89 | -1.06 | -0.73 | <0.001 |
| Saint Lucia | Alcohol use | -0.74 | -0.88 | -0.59 | <0.001 |
| Saint Vincent | All cause | 0.53 | 0.32 | 0.74 | <0.001 |
| Saint Vincent | Smoking | 0.48 | 0.26 | 0.7 | <0.001 |
| Saint Vincent | Alcohol use | 1.53 | 1.27 | 1.78 | <0.001 |
| Samoa | All cause | -0.9 | -0.95 | -0.85 | <0.001 |
| Samoa | Smoking | -1.14 | -1.19 | -1.1 | <0.001 |
| Samoa | Alcohol use | -1.08 | -1.41 | -0.76 | <0.001 |
| San Marino | All cause | -1.73 | -1.99 | -1.47 | <0.001 |
| San Marino | Smoking | -2.12 | -2.4 | -1.84 | <0.001 |
| San Marino | Alcohol use | -1.56 | -1.83 | -1.28 | <0.001 |
| Sao Tome and Principe | All cause | 0.95 | 0.83 | 1.06 | <0.001 |
| Sao Tome and Principe | Smoking | 1.36 | 1.14 | 1.58 | <0.001 |
| Sao Tome and Principe | Alcohol use | 1.67 | 1.38 | 1.96 | <0.001 |
| Saudi Arabia | All cause | -1.38 | -1.55 | -1.21 | <0.001 |
| Saudi Arabia | Smoking | -1.26 | -1.44 | -1.08 | <0.001 |
| Saudi Arabia | Alcohol use | -1.51 | -1.93 | -1.09 | <0.001 |
| Senegal | All cause | 0.44 | 0.16 | 0.72 | 0.003 |
| Senegal | Smoking | -0.32 | -0.59 | -0.04 | 0.027 |
| Senegal | Alcohol use | -0.36 | -0.74 | 0.01 | 0.058 |
| Serbia | All cause | -1.32 | -1.59 | -1.05 | <0.001 |
| Serbia | Smoking | -1.32 | -1.62 | -1.02 | <0.001 |
| Serbia | Alcohol use | -1.36 | -1.61 | -1.11 | <0.001 |
| Seychelles | All cause | -0.28 | -0.62 | 0.06 | 0.1 |
| Seychelles | Smoking | -0.37 | -0.69 | -0.05 | 0.024 |
| Seychelles | Alcohol use | 0.78 | 0.56 | 1.01 | <0.001 |
| Sierra Leone | All cause | 0.36 | 0.14 | 0.58 | 0.002 |
| Sierra Leone | Smoking | -0.02 | -0.27 | 0.22 | 0.842 |
| Sierra Leone | Alcohol use | -0.2 | -0.38 | -0.03 | 0.026 |
| Singapore | All cause | -5.18 | -5.45 | -4.9 | <0.001 |
| Singapore | Smoking | -5.84 | -6.09 | -5.59 | <0.001 |
| Singapore | Alcohol use | -4.69 | -5.09 | -4.28 | <0.001 |
| Slovakia | All cause | -2.67 | -2.78 | -2.57 | <0.001 |
| Slovakia | Smoking | -2.89 | -3 | -2.77 | <0.001 |
| Slovakia | Alcohol use | -2.92 | -3.06 | -2.78 | <0.001 |
| Slovenia | All cause | -3.02 | -3.2 | -2.83 | <0.001 |
| Slovenia | Smoking | -3.04 | -3.21 | -2.87 | <0.001 |
| Slovenia | Alcohol use | -4.2 | -4.42 | -3.99 | <0.001 |
| Solomon Islands | All cause | -0.4 | -0.46 | -0.35 | <0.001 |
| Solomon Islands | Smoking | -0.53 | -0.58 | -0.48 | <0.001 |
| Solomon Islands | Alcohol use | 1.39 | 1.09 | 1.69 | <0.001 |
| Somalia | All cause | -0.88 | -0.91 | -0.85 | <0.001 |
| Somalia | Smoking | -1.13 | -1.17 | -1.1 | <0.001 |
| Somalia | Alcohol use | 0 | 0 | 0 | 0.094 |
| South Africa | All cause | -1.66 | -2.23 | -1.09 | <0.001 |
| South Africa | Smoking | -2.34 | -2.9 | -1.77 | <0.001 |
| South Africa | Alcohol use | -1.88 | -2.47 | -1.29 | <0.001 |
| South Asia | All cause | -1.17 | -1.26 | -1.07 | <0.001 |
| South Asia | Smoking | -1.71 | -1.79 | -1.63 | <0.001 |
| South Asia | Alcohol use | 0.64 | 0.37 | 0.91 | <0.001 |
| South Korea | All cause | -6.52 | -7.15 | -5.89 | <0.001 |
| South Korea | Smoking | -6.51 | -7.13 | -5.9 | <0.001 |
| South Korea | Alcohol use | -6.02 | -6.63 | -5.41 | <0.001 |
| South Sudan | All cause | -1.2 | -1.26 | -1.14 | <0.001 |
| South Sudan | Smoking | -1.48 | -1.55 | -1.41 | <0.001 |
| South Sudan | Alcohol use | -1.09 | -1.31 | -0.87 | <0.001 |
| Southeast Asia | All cause | -0.66 | -0.74 | -0.57 | <0.001 |
| Southeast Asia | Smoking | -0.79 | -0.88 | -0.71 | <0.001 |
| Southeast Asia | Alcohol use | 1.86 | 1.68 | 2.03 | <0.001 |
| Southern Latin America | All cause | -2.36 | -2.53 | -2.19 | <0.001 |
| Southern Latin America | Smoking | -2.77 | -2.97 | -2.57 | <0.001 |
| Southern Latin America | Alcohol use | -2.88 | -3.03 | -2.72 | <0.001 |
| Southern Sub-Saharan Africa | All cause | -1.21 | -1.68 | -0.74 | <0.001 |
| Southern Sub-Saharan Africa | Smoking | -1.85 | -2.31 | -1.39 | <0.001 |
| Southern Sub-Saharan Africa | Alcohol use | -1.49 | -1.99 | -0.99 | <0.001 |
| Spain | All cause | -3.62 | -3.76 | -3.48 | <0.001 |
| Spain | Smoking | -3.97 | -4.1 | -3.85 | <0.001 |
| Spain | Alcohol use | -4.15 | -4.31 | -3.99 | <0.001 |
| Sri Lanka | All cause | 1.46 | 1.03 | 1.89 | <0.001 |
| Sri Lanka | Smoking | 0.26 | -0.14 | 0.67 | 0.188 |
| Sri Lanka | Alcohol use | 2.67 | 2.05 | 3.31 | <0.001 |
| Sudan | All cause | -0.27 | -0.32 | -0.22 | <0.001 |
| Sudan | Smoking | -0.32 | -0.4 | -0.24 | <0.001 |
| Sudan | Alcohol use | -7.37 | -9.41 | -5.28 | <0.001 |
| Suriname | All cause | 0.13 | -0.19 | 0.46 | 0.398 |
| Suriname | Smoking | -0.12 | -0.45 | 0.22 | 0.491 |
| Suriname | Alcohol use | 0.3 | 0.02 | 0.59 | 0.037 |
| Swaziland | All cause | 0.09 | -0.36 | 0.54 | 0.699 |
| Swaziland | Smoking | -0.81 | -1.21 | -0.42 | <0.001 |
| Swaziland | Alcohol use | 0.04 | -0.42 | 0.51 | 0.845 |
| Sweden | All cause | -1.56 | -1.65 | -1.48 | <0.001 |
| Sweden | Smoking | -1.88 | -1.97 | -1.79 | <0.001 |
| Sweden | Alcohol use | -1.42 | -1.54 | -1.29 | <0.001 |
| Switzerland | All cause | -3.19 | -3.46 | -2.91 | <0.001 |
| Switzerland | Smoking | -3.41 | -3.69 | -3.13 | <0.001 |
| Switzerland | Alcohol use | -3.42 | -3.69 | -3.15 | <0.001 |
| Syria | All cause | -0.94 | -1.26 | -0.61 | <0.001 |
| Syria | Smoking | -1.08 | -1.4 | -0.77 | <0.001 |
| Syria | Alcohol use | -2 | -2.25 | -1.76 | <0.001 |
| Taiwan | All cause | -2.35 | -2.65 | -2.05 | <0.001 |
| Taiwan | Smoking | -2.58 | -2.9 | -2.26 | <0.001 |
| Taiwan | Alcohol use | -3.51 | -3.9 | -3.12 | <0.001 |
| Tajikistan | All cause | -1.84 | -2.08 | -1.6 | <0.001 |
| Tajikistan | Smoking | -2.76 | -3.02 | -2.49 | <0.001 |
| Tajikistan | Alcohol use | -4.47 | -4.93 | -4.01 | <0.001 |
| Tanzania | All cause | -0.92 | -0.96 | -0.87 | <0.001 |
| Tanzania | Smoking | -1.33 | -1.4 | -1.26 | <0.001 |
| Tanzania | Alcohol use | -0.8 | -0.85 | -0.75 | <0.001 |
| Thailand | All cause | -2.54 | -2.77 | -2.3 | <0.001 |
| Thailand | Smoking | -2.85 | -3.1 | -2.6 | <0.001 |
| Thailand | Alcohol use | -1.78 | -1.99 | -1.57 | <0.001 |
| Timor-Leste | All cause | -0.44 | -0.75 | -0.12 | 0.008 |
| Timor-Leste | Smoking | -0.36 | -0.67 | -0.06 | 0.022 |
| Timor-Leste | Alcohol use | 2.43 | 2.11 | 2.75 | <0.001 |
| Togo | All cause | 0.56 | 0.41 | 0.71 | <0.001 |
| Togo | Smoking | -0.03 | -0.18 | 0.12 | 0.702 |
| Togo | Alcohol use | 0.35 | 0.03 | 0.66 | 0.032 |
| Tokelau | All cause | -1.29 | -1.35 | -1.23 | <0.001 |
| Tokelau | Smoking | -1.49 | -1.55 | -1.44 | <0.001 |
| Tokelau | Alcohol use | -0.33 | -0.38 | -0.28 | <0.001 |
| Tonga | All cause | -0.57 | -0.82 | -0.32 | <0.001 |
| Tonga | Smoking | -0.79 | -1.06 | -0.52 | <0.001 |
| Tonga | Alcohol use | 0.84 | 0.09 | 1.6 | 0.03 |
| Trinidad | All cause | -1.83 | -2.07 | -1.6 | <0.001 |
| Trinidad | Smoking | -2.09 | -2.31 | -1.87 | <0.001 |
| Trinidad | Alcohol use | -0.85 | -1.06 | -0.64 | <0.001 |
| Tropical Latin America | All cause | -0.92 | -1 | -0.85 | <0.001 |
| Tropical Latin America | Smoking | -2.02 | -2.17 | -1.86 | <0.001 |
| Tropical Latin America | Alcohol use | -0.54 | -0.8 | -0.29 | <0.001 |
| Tunisia | All cause | -0.87 | -0.98 | -0.76 | <0.001 |
| Tunisia | Smoking | -0.96 | -1.08 | -0.85 | <0.001 |
| Tunisia | Alcohol use | 1.33 | 1.2 | 1.47 | <0.001 |
| Turkey | All cause | -1.82 | -2.13 | -1.51 | <0.001 |
| Turkey | Smoking | -2.1 | -2.39 | -1.81 | <0.001 |
| Turkey | Alcohol use | -2.17 | -2.41 | -1.92 | <0.001 |
| Turkmenistan | All cause | -4.14 | -4.62 | -3.66 | <0.001 |
| Turkmenistan | Smoking | -5.08 | -5.47 | -4.69 | <0.001 |
| Turkmenistan | Alcohol use | -2.38 | -2.79 | -1.96 | <0.001 |
| Tuvalu | All cause | -0.95 | -1.01 | -0.89 | <0.001 |
| Tuvalu | Smoking | -1.14 | -1.22 | -1.06 | <0.001 |
| Tuvalu | Alcohol use | -0.96 | -1.25 | -0.66 | <0.001 |
| Uganda | All cause | -0.96 | -1.18 | -0.74 | <0.001 |
| Uganda | Smoking | -1.48 | -1.78 | -1.17 | <0.001 |
| Uganda | Alcohol use | -0.55 | -0.8 | -0.3 | <0.001 |
| Ukraine | All cause | -3.07 | -3.56 | -2.57 | <0.001 |
| Ukraine | Smoking | -3.17 | -3.66 | -2.68 | <0.001 |
| Ukraine | Alcohol use | -2.06 | -2.45 | -1.67 | <0.001 |
| United Arab Emirates | All cause | -0.85 | -1.17 | -0.52 | <0.001 |
| United Arab Emirates | Smoking | -1.09 | -1.42 | -0.76 | <0.001 |
| United Arab Emirates | Alcohol use | -3.74 | -3.88 | -3.6 | <0.001 |
| United Kingdom | All cause | -1.77 | -1.89 | -1.65 | <0.001 |
| United Kingdom | Smoking | -2.29 | -2.39 | -2.18 | <0.001 |
| United Kingdom | Alcohol use | -1.62 | -1.76 | -1.48 | <0.001 |
| Uruguay | All cause | -2.16 | -2.32 | -2.01 | <0.001 |
| Uruguay | Smoking | -2.5 | -2.66 | -2.35 | <0.001 |
| Uruguay | Alcohol use | -2.1 | -2.29 | -1.9 | <0.001 |
| USA | All cause | -1.79 | -1.91 | -1.67 | <0.001 |
| USA | Smoking | -2.14 | -2.26 | -2.02 | <0.001 |
| USA | Alcohol use | -1.35 | -1.48 | -1.23 | <0.001 |
| Uzbekistan | All cause | -1.15 | -1.81 | -0.49 | 0.001 |
| Uzbekistan | Smoking | -2.04 | -2.67 | -1.41 | <0.001 |
| Uzbekistan | Alcohol use | -2.54 | -3.18 | -1.91 | <0.001 |
| Vanuatu | All cause | -0.59 | -0.73 | -0.45 | <0.001 |
| Vanuatu | Smoking | -1.1 | -1.24 | -0.97 | <0.001 |
| Vanuatu | Alcohol use | -0.63 | -1.25 | -0.01 | 0.046 |
| Venezuela | All cause | -0.78 | -1.07 | -0.49 | <0.001 |
| Venezuela | Smoking | -1.54 | -1.81 | -1.27 | <0.001 |
| Venezuela | Alcohol use | -1.33 | -1.68 | -0.99 | <0.001 |
| Vietnam | All cause | 0.94 | 0.88 | 1.01 | <0.001 |
| Vietnam | Smoking | 0.84 | 0.78 | 0.9 | <0.001 |
| Vietnam | Alcohol use | 9.75 | 8.93 | 10.58 | <0.001 |
| Virgin Islands | All cause | 1.4 | 0.99 | 1.81 | <0.001 |
| Virgin Islands | Smoking | 1.04 | 0.65 | 1.43 | <0.001 |
| Virgin Islands | Alcohol use | 1.89 | 1.42 | 2.37 | <0.001 |
| Western Europe | All cause | -2.84 | -2.95 | -2.72 | <0.001 |
| Western Europe | Smoking | -3.18 | -3.29 | -3.07 | <0.001 |
| Western Europe | Alcohol use | -3.15 | -3.27 | -3.02 | <0.001 |
| Western Sub-Saharan Africa | All cause | 0.22 | 0.08 | 0.36 | 0.003 |
| Western Sub-Saharan Africa | Smoking | -0.03 | -0.16 | 0.11 | 0.673 |
| Western Sub-Saharan Africa | Alcohol use | 0.59 | 0.41 | 0.78 | <0.001 |
| Yemen | All cause | 0.08 | 0.03 | 0.14 | 0.005 |
| Yemen | Smoking | 0.11 | 0.06 | 0.16 | <0.001 |
| Yemen | Alcohol use | -3.16 | -3.48 | -2.83 | <0.001 |
| Zambia | All cause | -0.96 | -1.23 | -0.69 | <0.001 |
| Zambia | Smoking | -0.96 | -1.26 | -0.66 | <0.001 |
| Zambia | Alcohol use | -0.23 | -0.3 | -0.16 | <0.001 |
| Zimbabwe | All cause | -0.01 | -0.13 | 0.11 | 0.801 |
| Zimbabwe | Smoking | -0.47 | -0.59 | -0.35 | <0.001 |
| Zimbabwe | Alcohol use | -0.83 | -1.07 | -0.59 | <0.001 |

Table S3. The temporal trends of alcohol consumption at the national level.

| Location name | EAPC | 95% CI | | P value |
| --- | --- | --- | --- | --- |
| Afghanistan | 3.63 | 2.48 | 4.8 | <0.001 |
| Albania | 4.72 | 3.82 | 5.63 | <0.001 |
| Algeria | 1.27 | 0.18 | 2.37 | 0.024 |
| Andorra | 7.72 | 5.77 | 9.71 | <0.001 |
| Angola | 2.32 | 1.21 | 3.44 | <0.001 |
| Antigua and Barbuda | -2.12 | -2.49 | -1.75 | <0.001 |
| Argentina | 6.11 | 4.13 | 8.12 | <0.001 |
| Armenia | -4.8 | -5.74 | -3.85 | <0.001 |
| Australia | -1.23 | -1.47 | -0.98 | <0.001 |
| Austria | 10.57 | 6.16 | 15.15 | <0.001 |
| Azerbaijan | -10.17 | -12.83 | -7.42 | <0.001 |
| Bahamas | 2.59 | 1.47 | 3.72 | <0.001 |
| Bahrain | 24.21 | 18.54 | 30.14 | <0.001 |
| Bangladesh | -30.64 | -37.73 | -22.75 | <0.001 |
| Barbados | -0.71 | -1.48 | 0.07 | 0.072 |
| Belarus | 0.32 | -0.11 | 0.76 | 0.144 |
| Belgium | 2.36 | 1.46 | 3.27 | <0.001 |
| Belize | 6.37 | 4.32 | 8.46 | <0.001 |
| Benin | 3.79 | 1.54 | 6.1 | 0.002 |
| Bhutan | -10.51 | -12.93 | -8.01 | <0.001 |
| Bolivia | -5.52 | -7.84 | -3.15 | <0.001 |
| Bosnia and Herzegovina | 0.55 | 0.07 | 1.04 | 0.027 |
| Botswana | 1.84 | 1.37 | 2.32 | <0.001 |
| Brazil | 4.25 | 0.65 | 7.97 | 0.022 |
| Brunei Darussalam | -13.41 | -16.92 | -9.75 | <0.001 |
| Bulgaria | 4.68 | 3.98 | 5.39 | <0.001 |
| Burkina Faso | -2.54 | -3.27 | -1.81 | <0.001 |
| Burundi | 1.61 | 0.68 | 2.56 | 0.001 |
| Cote d'Ivoire | 0.72 | 0 | 1.44 | 0.05 |
| Cabo Verde | 13.83 | 11.26 | 16.45 | <0.001 |
| Cambodia | -5.04 | -6.01 | -4.06 | <0.001 |
| Cameroon | -2.59 | -3.19 | -1.99 | <0.001 |
| Canada | 4.87 | 3.27 | 6.48 | <0.001 |
| Central African Republic | 6.96 | 5.03 | 8.93 | <0.001 |
| Chad | -12.08 | -14.57 | -9.51 | <0.001 |
| Chile | 4.53 | 4.04 | 5.03 | <0.001 |
| China | -1.52 | -2.46 | -0.57 | 0.003 |
| Colombia | 15.22 | 10.86 | 19.75 | <0.001 |
| Comoros | -13.46 | -15.8 | -11.06 | <0.001 |
| Congo | -0.21 | -2.66 | 2.31 | 0.864 |
| Cook Islands | 1.18 | -0.38 | 2.77 | 0.133 |
| Costa Rica | -6.19 | -7.41 | -4.96 | <0.001 |
| Croatia | 5.21 | 4.01 | 6.42 | <0.001 |
| Cuba | -2.62 | -3.45 | -1.78 | <0.001 |
| Cyprus | -0.99 | -1.31 | -0.68 | <0.001 |
| Czech Republic | 6.01 | 5.13 | 6.89 | <0.001 |
| North Korea | 1.69 | 1.1 | 2.29 | <0.001 |
| Democratic Republic of the Congo | -8.1 | -9.97 | -6.19 | <0.001 |
| Denmark | 8.16 | 4.89 | 11.53 | <0.001 |
| Djibouti | -15.56 | -17.84 | -13.21 | <0.001 |
| Dominica | 3.13 | 2.5 | 3.75 | <0.001 |
| Dominican Republic | 3.36 | 2.05 | 4.69 | <0.001 |
| Ecuador | 11.31 | 6.84 | 15.96 | <0.001 |
| Egypt | -9.89 | -12.13 | -7.58 | <0.001 |
| El Salvador | 4.16 | 0.32 | 8.15 | 0.034 |
| Equatorial Guinea | 12.95 | 10.8 | 15.14 | <0.001 |
| Eritrea | -0.28 | -1.08 | 0.53 | 0.481 |
| Estonia | 8.47 | 5.99 | 11.01 | <0.001 |
| Ethiopia | -7.85 | -9.93 | -5.73 | <0.001 |
| Fiji | -9.1 | -11 | -7.16 | <0.001 |
| Finland | -1.03 | -1.54 | -0.53 | <0.001 |
| France | 9.45 | 7.48 | 11.47 | <0.001 |
| Gabon | 4.41 | 1.87 | 7.02 | 0.001 |
| The Gambia | -7.26 | -8.92 | -5.57 | <0.001 |
| Georgia | 5.84 | 4.78 | 6.92 | <0.001 |
| Germany | 0.16 | -0.18 | 0.49 | 0.348 |
| Ghana | -8.6 | -10.89 | -6.26 | <0.001 |
| Greece | 4.94 | 3.26 | 6.66 | <0.001 |
| Grenada | 20.18 | 17.03 | 23.42 | <0.001 |
| Guatemala | 0.18 | -3.05 | 3.52 | 0.91 |
| Guinea | -14.12 | -17.29 | -10.83 | <0.001 |
| Guinea-Bissau | -3.01 | -3.87 | -2.13 | <0.001 |
| Guyana | 5.09 | 4.09 | 6.1 | <0.001 |
| Haiti | -4.3 | -4.97 | -3.63 | <0.001 |
| Honduras | -2.13 | -2.65 | -1.61 | <0.001 |
| Hungary | 9.93 | 8.14 | 11.75 | <0.001 |
| Iceland | 20.91 | 16.38 | 25.62 | <0.001 |
| India | 25.28 | 20.07 | 30.71 | <0.001 |
| Indonesia | -8.33 | -9.64 | -7 | <0.001 |
| Iran | -23.5 | -28.91 | -17.69 | <0.001 |
| Iraq | -11.41 | -13.83 | -8.93 | <0.001 |
| Ireland | -0.36 | -0.91 | 0.19 | 0.194 |
| Israel | -1.45 | -1.95 | -0.95 | <0.001 |
| Italy | 0.31 | -0.03 | 0.65 | 0.07 |
| Jamaica | 10.94 | 7.39 | 14.6 | <0.001 |
| Japan | -0.41 | -2.73 | 1.97 | 0.722 |
| Jordan | -6.99 | -7.93 | -6.03 | <0.001 |
| Kazakhstan | 10.1 | 7.67 | 12.59 | <0.001 |
| Kenya | 24.76 | 18.91 | 30.89 | <0.001 |
| Kiribati | -0.45 | -2.27 | 1.41 | 0.621 |
| Kuwait | 2.79 | -3.43 | 9.42 | 0.375 |
| Kyrgyzstan | 1.87 | 0.66 | 3.1 | 0.004 |
| Lao PDR | 10.77 | 7.68 | 13.96 | <0.001 |
| Latvia | 9.96 | 7.44 | 12.54 | <0.001 |
| Lebanon | 5.13 | 2.73 | 7.59 | <0.001 |
| Lesotho | 27.77 | 20.6 | 35.37 | <0.001 |
| Liberia | -1.8 | -2.1 | -1.5 | <0.001 |
| Libya | -20.79 | -26.95 | -14.11 | <0.001 |
| Lithuania | 2.48 | 1.62 | 3.34 | <0.001 |
| Luxembourg | 19.17 | 13.88 | 24.7 | <0.001 |
| Madagascar | 17.87 | 13.42 | 22.49 | <0.001 |
| Malawi | 19.2 | 14.09 | 24.54 | <0.001 |
| Malaysia | 14.98 | 11.31 | 18.77 | <0.001 |
| Maldives | 2.08 | 0.94 | 3.23 | 0.001 |
| Mali | 1.96 | 1.28 | 2.65 | <0.001 |
| Malta | 27.15 | 20.32 | 34.36 | <0.001 |
| Marshall Islands | 9.6 | 7.63 | 11.62 | <0.001 |
| Mauritania | -4.71 | -5.7 | -3.72 | <0.001 |
| Mauritius | 29.18 | 21.15 | 37.75 | <0.001 |
| Mexico | 30.16 | 24.42 | 36.17 | <0.001 |
| Federated States of Micronesia | 15.87 | 11.85 | 20.03 | <0.001 |
| Monaco | 1.25 | -0.76 | 3.31 | 0.216 |
| Mongolia | 1.25 | -0.76 | 3.31 | 0.216 |
| Montenegro | 11.88 | 7.67 | 16.25 | <0.001 |
| Morocco | 7.14 | 5.18 | 9.12 | <0.001 |
| Mozambique | 13.34 | 11.66 | 15.05 | <0.001 |
| Myanmar | -8.81 | -10.4 | -7.18 | <0.001 |
| Namibia | -13.8 | -16.4 | -11.12 | <0.001 |
| Nauru | 2.95 | 1.89 | 4.03 | <0.001 |
| Nepal | 7.84 | 3.34 | 12.55 | 0.001 |
| Netherlands | -19.7 | -25.41 | -13.55 | <0.001 |
| New Zealand | 0.77 | 0.43 | 1.12 | <0.001 |
| Nicaragua | 1.41 | 0.62 | 2.2 | 0.001 |
| Niger | -1.51 | -1.83 | -1.19 | <0.001 |
| Nigeria | -3.66 | -5.82 | -1.46 | 0.002 |
| Niue | 27.85 | 21.46 | 34.57 | <0.001 |
| Norway | 1.23 | 0.6 | 1.85 | <0.001 |
| Oman | 6.11 | 5.11 | 7.13 | <0.001 |
| Pakistan | -10.35 | -12.77 | -7.85 | <0.001 |
| Panama | -19.21 | -23.11 | -15.12 | <0.001 |
| Papua New Guinea | 2.15 | 1.71 | 2.59 | <0.001 |
| Paraguay | -10.46 | -12.78 | -8.08 | <0.001 |
| Peru | -3.95 | -4.53 | -3.37 | <0.001 |
| Philippines | 3.5 | 1.01 | 6.05 | 0.007 |
| Poland | -3.64 | -4.13 | -3.16 | <0.001 |
| Portugal | 2.58 | 1.71 | 3.46 | <0.001 |
| Qatar | -1.19 | -2.09 | -0.29 | 0.012 |
| South Korea | -8.87 | -10.95 | -6.75 | <0.001 |
| Moldova | 1.2 | 0.54 | 1.86 | 0.001 |
| Romania | 1.07 | 0.51 | 1.63 | <0.001 |
| Russian Federation | 3.67 | 3 | 4.33 | <0.001 |
| Rwanda | 0.5 | 0.15 | 0.86 | 0.007 |
| Saint Kitts and Nevis | 1.14 | 0.6 | 1.67 | <0.001 |
| Saint Lucia | 7.45 | 5.94 | 8.99 | <0.001 |
| Saint Vincent and the Grenadines | -1.62 | -2.48 | -0.75 | 0.001 |
| Samoa | 3.34 | 2.64 | 4.04 | <0.001 |
| San Marino | 28.99 | 21.16 | 37.32 | <0.001 |
| Sao Tome and Principe | 0 | 0 | 0 | 0.100 |
| Saudi Arabia | 31.59 | 23.07 | 40.7 | <0.001 |
| Senegal | 11.56 | 8.29 | 14.93 | <0.001 |
| Serbia | -10.47 | -12.75 | -8.13 | <0.001 |
| Seychelles | 22.2 | 15.35 | 29.46 | <0.001 |
| Sierra Leone | 33.52 | 24.16 | 43.59 | <0.001 |
| Singapore | 30.54 | 25.39 | 35.91 | <0.001 |
| Slovakia | 23.73 | 18.42 | 29.29 | <0.001 |
| Slovenia | 33.9 | 25.82 | 42.5 | <0.001 |
| Solomon Islands | 7.67 | 5.47 | 9.92 | <0.001 |
| Somalia | 22.58 | 16.79 | 28.65 | <0.001 |
| South Africa | 0 | 0 | 0 | 0.094 |
| Spain | 27.46 | 21.57 | 33.62 | <0.001 |
| Sri Lanka | 34.6 | 25.94 | 43.87 | <0.001 |
| Sudan | -2.65 | -3.85 | -1.44 | <0.001 |
| Suriname | 25.84 | 19.12 | 32.94 | <0.001 |
| Swaziland | 12.77 | 9.02 | 16.66 | <0.001 |
| Sweden | 31.85 | 23.17 | 41.14 | <0.001 |
| Switzerland | 1.05 | 0.64 | 1.46 | <0.001 |
| Syria | 33.75 | 25.66 | 42.37 | <0.001 |
| Tajikistan | 37.57 | 22.95 | 53.93 | <0.001 |
| Thailand | 27.28 | 17.91 | 37.39 | <0.001 |
| The former Yugoslav republic of Macedonia | 19.2 | 13.73 | 24.93 | <0.001 |
| Timor-Leste | 32.29 | 20.89 | 44.76 | <0.001 |
| Togo | 9.62 | 4.25 | 15.28 | 0.001 |
| Tonga | 23.56 | 17.54 | 29.88 | <0.001 |
| Trinidad and Tobago | 24.21 | 17.7 | 31.09 | <0.001 |
| Tunisia | 47.22 | 31.52 | 64.79 | <0.001 |
| Turkey | 32.69 | 23.14 | 42.99 | <0.001 |
| Turkmenistan | -6.69 | -8.06 | -5.3 | <0.001 |
| Tuvalu | 36.24 | 25.39 | 48.02 | <0.001 |
| Uganda | 23.13 | 16.94 | 29.64 | <0.001 |
| Ukraine | 29.83 | 25.04 | 34.81 | <0.001 |
| United Arab Emirates | 0.85 | 0.02 | 1.69 | 0.046 |
| United Kingdom | 0.54 | 0.1 | 0.97 | 0.020 |
| Tanzania | 15.46 | 10.82 | 20.3 | <0.001 |
| United States of America | 1.39 | -0.81 | 3.63 | 0.195 |
| Uruguay | 40.82 | 28.3 | 54.56 | <0.001 |
| Uzbekistan | -0.14 | -1.19 | 0.93 | 0.786 |
| Vanuatu | 6.65 | 5.17 | 8.15 | <0.001 |
| Venezuela | 36.2 | 24.7 | 48.76 | <0.001 |
| Viet Nam | 5.15 | 3.87 | 6.45 | <0.001 |
| Yemen | 3.56 | 2.15 | 4.98 | <0.001 |
| Zambia | -3.68 | -6.32 | -0.97 | 0.012 |
| Zimbabwe | -0.22 | -1.3 | 0.88 | 0.673 |

Table S4. The temporal trends of smoking prevalence at the national level.

| Country | EAPC | 95% CI | | P value |
| --- | --- | --- | --- | --- |
| Afghanistan | 0.52 | 0.47 | 0.58 | <0.001 |
| Albania | -0.43 | -0.56 | -0.29 | <0.001 |
| Algeria | -1.17 | -1.52 | -0.81 | <0.001 |
| Andorra | -0.47 | -0.58 | -0.35 | <0.001 |
| Angola | 0 | -0.03 | 0.03 | 1.000 |
| Antigua and Barbuda | -1.29 | -1.46 | -1.13 | <0.001 |
| Argentina | -0.76 | -0.88 | -0.65 | <0.001 |
| Armenia | -0.1 | -0.2 | -0.01 | 0.033 |
| Australia | -1.98 | -2.06 | -1.89 | <0.001 |
| Austria | 0.56 | 0.41 | 0.7 | <0.001 |
| Azerbaijan | 0.09 | -0.01 | 0.19 | 0.079 |
| Bahamas | -0.37 | -0.46 | -0.28 | <0.001 |
| Bahrain | -0.01 | -0.49 | 0.49 | 0.981 |
| Bangladesh | -0.26 | -0.31 | -0.21 | <0.001 |
| Barbados | -0.49 | -0.56 | -0.41 | <0.001 |
| Belarus | -0.41 | -0.61 | -0.22 | <0.001 |
| Belgium | -0.88 | -1.02 | -0.74 | <0.001 |
| Belize | -0.8 | -0.87 | -0.73 | <0.001 |
| Benin | -0.59 | -0.65 | -0.53 | <0.001 |
| Bhutan | -0.31 | -0.43 | -0.19 | <0.001 |
| Bolivia | -0.9 | -1.22 | -0.59 | <0.001 |
| Bosnia and Herzegovina | -0.61 | -0.74 | -0.47 | <0.001 |
| Botswana | -0.47 | -0.54 | -0.39 | <0.001 |
| Brazil | -1.01 | -1.1 | -0.93 | <0.001 |
| Brunei | -0.07 | -0.15 | 0.01 | 0.091 |
| Bulgaria | 0.94 | 0.68 | 1.21 | <0.001 |
| Burkina Faso | -0.68 | -0.79 | -0.57 | <0.001 |
| Burundi | -0.4 | -0.53 | -0.27 | <0.001 |
| Cambodia | -0.54 | -0.71 | -0.38 | <0.001 |
| Cameroon | -0.62 | -0.71 | -0.54 | <0.001 |
| Canada | -3.22 | -3.39 | -3.04 | <0.001 |
| Cape Verde | 0.27 | 0.24 | 0.31 | <0.001 |
| Central African Republic | -0.04 | -0.06 | -0.01 | 0.004 |
| Chad | -0.21 | -0.23 | -0.2 | <0.001 |
| Chile | -0.86 | -1.12 | -0.61 | <0.001 |
| China | -0.78 | -0.94 | -0.62 | <0.001 |
| Colombia | -1.15 | -1.2 | -1.09 | <0.001 |
| Comoros | -0.56 | -0.77 | -0.36 | <0.001 |
| Congo | 0.02 | -0.04 | 0.09 | 0.449 |
| Costa Rica | 0.5 | 0.23 | 0.76 | 0.001 |
| Cote d'Ivoire | 0.06 | -0.14 | 0.26 | 0.548 |
| Croatia | 0.16 | 0.09 | 0.24 | <0.001 |
| Cuba | -0.54 | -0.7 | -0.38 | <0.001 |
| Cyprus | -0.27 | -0.36 | -0.19 | <0.001 |
| Czech Republic | -0.18 | -0.26 | -0.09 | <0.001 |
| Democratic Republic of Congo | 0.01 | 0 | 0.01 | 0.093 |
| Denmark | -2.14 | -2.49 | -1.79 | <0.001 |
| Djibouti | 0.03 | -0.01 | 0.07 | 0.142 |
| Dominica | -0.47 | -0.52 | -0.43 | <0.001 |
| Dominican Republic | -1.53 | -1.86 | -1.2 | <0.001 |
| Ecuador | -1.38 | -1.69 | -1.07 | <0.001 |
| Egypt | 0.19 | 0.05 | 0.33 | 0.008 |
| El Salvador | -0.66 | -0.73 | -0.59 | <0.001 |
| Equatorial Guinea | -0.03 | -0.39 | 0.32 | 0.851 |
| Eritrea | -0.72 | -0.81 | -0.63 | <0.001 |
| Estonia | 0.04 | -0.11 | 0.19 | 0.574 |
| Ethiopia | -0.65 | -0.71 | -0.58 | <0.001 |
| Fiji | -0.55 | -0.63 | -0.48 | <0.001 |
| Finland | -1.21 | -1.4 | -1.03 | <0.001 |
| France | -0.56 | -0.78 | -0.34 | <0.001 |
| Gabon | 0.59 | 0.48 | 0.7 | <0.001 |
| Gambia | -0.97 | -1.04 | -0.9 | <0.001 |
| Georgia | -0.22 | -0.29 | -0.16 | <0.001 |
| Germany | -1.2 | -1.29 | -1.11 | <0.001 |
| Ghana | -0.91 | -1.05 | -0.76 | <0.001 |
| Greece | -0.04 | -0.16 | 0.09 | 0.556 |
| Grenada | 0.36 | 0.2 | 0.51 | <0.001 |
| Guatemala | -1.02 | -1.27 | -0.78 | <0.001 |
| Guinea | -0.32 | -0.36 | -0.28 | <0.001 |
| Guinea-Bissau | -0.6 | -0.69 | -0.51 | <0.001 |
| Guyana | 1.07 | 0.94 | 1.19 | <0.001 |
| Haiti | -1.04 | -1.19 | -0.88 | <0.001 |
| Honduras | -1.24 | -1.32 | -1.15 | <0.001 |
| Hungary | -0.24 | -0.4 | -0.08 | 0.004 |
| Iceland | -2.86 | -3.11 | -2.62 | <0.001 |
| India | -1.06 | -1.23 | -0.88 | <0.001 |
| Indonesia | 0.13 | 0.05 | 0.21 | 0.002 |
| Iran | -0.23 | -0.29 | -0.16 | <0.001 |
| Iraq | -0.23 | -0.28 | -0.18 | <0.001 |
| Ireland | -1.46 | -1.61 | -1.31 | <0.001 |
| Israel | -1.92 | -2.04 | -1.79 | <0.001 |
| Italy | -1.35 | -1.46 | -1.24 | <0.001 |
| Jamaica | -0.1 | -0.2 | 0.01 | 0.073 |
| Japan | -1.59 | -1.75 | -1.42 | <0.001 |
| Jordan | -0.27 | -0.35 | -0.18 | <0.001 |
| Kazakhstan | 0.39 | 0.16 | 0.62 | 0.002 |
| Kenya | -0.54 | -0.69 | -0.4 | <0.001 |
| Kiribati | -0.3 | -0.5 | -0.09 | 0.007 |
| Kuwait | -0.49 | -0.9 | -0.07 | 0.024 |
| Kyrgyzstan | -0.78 | -0.97 | -0.59 | <0.001 |
| Laos | -0.75 | -0.9 | -0.61 | <0.001 |
| Latvia | 0.22 | 0.14 | 0.3 | <0.001 |
| Lebanon | -1.21 | -1.51 | -0.91 | <0.001 |
| Lesotho | -0.75 | -0.94 | -0.56 | <0.001 |
| Liberia | -0.54 | -0.62 | -0.45 | <0.001 |
| Libya | -0.18 | -0.77 | 0.41 | 0.535 |
| Lithuania | 0.88 | 0.77 | 0.99 | <0.001 |
| Luxembourg | -0.8 | -0.87 | -0.73 | <0.001 |
| Macedonia | 0.12 | 0.06 | 0.19 | 0.001 |
| Madagascar | -1.51 | -1.86 | -1.16 | <0.001 |
| Malawi | -0.99 | -1.2 | -0.77 | <0.001 |
| Malaysia | -0.78 | -0.87 | -0.7 | <0.001 |
| Maldives | -0.25 | -0.38 | -0.13 | <0.001 |
| Mali | -0.02 | -0.06 | 0.01 | 0.164 |
| Malta | -0.89 | -1.12 | -0.65 | <0.001 |
| Marshall Islands | -0.29 | -0.34 | -0.24 | <0.001 |
| Mauritania | 1.24 | 1.16 | 1.32 | <0.001 |
| Mauritius | -0.82 | -0.99 | -0.65 | <0.001 |
| Mexico | -3.95 | -4.42 | -3.48 | <0.001 |
| Micronesia | -0.28 | -0.31 | -0.25 | <0.001 |
| Moldova | -1.27 | -1.5 | -1.05 | <0.001 |
| Mongolia | 0.51 | 0.44 | 0.57 | <0.001 |
| Montenegro | -0.01 | -0.06 | 0.04 | 0.697 |
| Morocco | -0.02 | -0.07 | 0.02 | 0.297 |
| Mozambique | -0.24 | -0.32 | -0.17 | <0.001 |
| Myanmar | -0.73 | -0.85 | -0.61 | <0.001 |
| Namibia | -0.74 | -0.94 | -0.55 | <0.001 |
| Nepal | -1.12 | -1.48 | -0.75 | <0.001 |
| Netherlands | -0.99 | -1.14 | -0.84 | <0.001 |
| New Zealand | -2.01 | -2.21 | -1.82 | <0.001 |
| Nicaragua | -1.33 | -1.55 | -1.1 | <0.001 |
| Niger | -0.29 | -0.38 | -0.2 | <0.001 |
| Nigeria | -3.31 | -3.66 | -2.97 | <0.001 |
| North Korea | 0.23 | 0.18 | 0.29 | <0.001 |
| Norway | -2.37 | -2.71 | -2.04 | <0.001 |
| Oman | -1.17 | -1.66 | -0.69 | <0.001 |
| Pakistan | -0.81 | -0.92 | -0.7 | <0.001 |
| Palestine | -0.64 | -0.81 | -0.47 | <0.001 |
| Panama | -0.84 | -0.97 | -0.71 | <0.001 |
| Papua New Guinea | -0.01 | -0.03 | 0.02 | 0.472 |
| Paraguay | -1.11 | -1.27 | -0.95 | <0.001 |
| Peru | 0.47 | 0.41 | 0.53 | <0.001 |
| Philippines | -0.79 | -0.98 | -0.59 | <0.001 |
| Poland | -1.3 | -1.38 | -1.22 | <0.001 |
| Portugal | 0.11 | -0.01 | 0.23 | 0.079 |
| Qatar | 0.41 | 0.25 | 0.57 | <0.001 |
| Romania | 0 | -0.21 | 0.2 | 0.966 |
| Russia | -0.49 | -0.6 | -0.37 | <0.001 |
| Rwanda | -1.85 | -2.23 | -1.47 | <0.001 |
| Saint Lucia | 0.48 | 0.35 | 0.61 | <0.001 |
| Saint Vincent and the Grenadines | -0.06 | -0.1 | -0.02 | 0.003 |
| Samoa | -0.64 | -0.83 | -0.45 | <0.001 |
| Sao Tome and Principe | -0.49 | -0.63 | -0.35 | <0.001 |
| Saudi Arabia | 3.05 | 2.58 | 3.52 | <0.001 |
| Senegal | -1.45 | -1.67 | -1.22 | <0.001 |
| Serbia | 0.92 | 0.62 | 1.21 | <0.001 |
| Seychelles | -0.45 | -0.53 | -0.36 | <0.001 |
| Sierra Leone | -0.23 | -0.3 | -0.17 | <0.001 |
| Singapore | -0.98 | -1.03 | -0.92 | <0.001 |
| Slovakia | -0.27 | -0.35 | -0.18 | <0.001 |
| Slovenia | -1.19 | -1.29 | -1.08 | <0.001 |
| Solomon Islands | -0.24 | -0.28 | -0.2 | <0.001 |
| Somalia | -0.58 | -0.64 | -0.51 | <0.001 |
| South Africa | -1.8 | -2.09 | -1.51 | <0.001 |
| South Korea | -1.22 | -1.38 | -1.05 | <0.001 |
| Spain | -1.17 | -1.33 | -1 | <0.001 |
| Sri Lanka | -0.5 | -0.56 | -0.43 | <0.001 |
| Sudan | -0.11 | -0.13 | -0.08 | <0.001 |
| Suriname | 0.01 | -0.06 | 0.08 | 0.701 |
| Swaziland | 0.52 | 0.37 | 0.67 | <0.001 |
| Sweden | -2.76 | -3.05 | -2.47 | <0.001 |
| Switzerland | -1.18 | -1.57 | -0.78 | <0.001 |
| Syria | -0.13 | -0.22 | -0.04 | 0.007 |
| Taiwan | -0.73 | -0.98 | -0.48 | <0.001 |
| Tajikistan | -1.16 | -1.51 | -0.8 | <0.001 |
| Tanzania | -0.65 | -0.77 | -0.53 | <0.001 |
| Thailand | -1.02 | -1.2 | -0.84 | <0.001 |
| Timor | -0.16 | -0.26 | -0.06 | 0.003 |
| Togo | -0.59 | -0.69 | -0.49 | <0.001 |
| Tonga | 0.38 | 0.26 | 0.49 | <0.001 |
| Trinidad and Tobago | -0.56 | -0.64 | -0.48 | <0.001 |
| Tunisia | 0.58 | 0.53 | 0.63 | <0.001 |
| Turkey | 0.15 | 0.02 | 0.29 | 0.028 |
| Turkmenistan | 0.07 | -0.09 | 0.23 | 0.392 |
| Uganda | -1.41 | -1.78 | -1.05 | <0.001 |
| Ukraine | -0.37 | -0.5 | -0.24 | <0.001 |
| United Arab Emirates | -0.99 | -1.18 | -0.8 | <0.001 |
| United Kingdom | -1.78 | -1.91 | -1.65 | <0.001 |
| United States | -2.1 | -2.14 | -2.06 | <0.001 |
| Uruguay | -1.04 | -1.22 | -0.85 | <0.001 |
| Uzbekistan | 0.14 | 0.02 | 0.27 | 0.029 |
| Vanuatu | -0.94 | -1.06 | -0.81 | <0.001 |
| Venezuela | -1.23 | -1.39 | -1.07 | <0.001 |
| Vietnam | -1.24 | -1.57 | -0.91 | <0.001 |
| World | -1.08 | -1.18 | -0.99 | <0.001 |
| Yemen | -1.35 | -1.66 | -1.04 | <0.001 |
| Zambia | -0.37 | -0.46 | -0.28 | <0.001 |
| Zimbabwe | -0.55 | -0.66 | -0.44 | 0 |
